# Synaptic balancing: A biologically plausible local learning rule that provably increases neural network noise robustness without sacrificing task performance

**DOI:** 10.1371/journal.pcbi.1010418

**Published:** 2022-09-19

**Authors:** Christopher H. Stock, Sarah E. Harvey, Samuel A. Ocko, Surya Ganguli

**Affiliations:** 1 Neuroscience Graduate Program, Stanford University School of Medicine, Stanford, California, United States of America; 2 Department of Applied Physics, Stanford University, Stanford, California, United States of America; 3 Stanford Institute for Human-Centered Artificial Intelligence, Stanford University, Stanford, California, United States of America; McGill University, CANADA

## Abstract

We introduce a novel, biologically plausible local learning rule that provably increases the robustness of neural dynamics to noise in nonlinear recurrent neural networks with homogeneous nonlinearities. Our learning rule achieves higher noise robustness without sacrificing performance on the task and without requiring any knowledge of the particular task. The plasticity dynamics—an integrable dynamical system operating on the weights of the network—maintains a multiplicity of conserved quantities, most notably the network’s entire temporal map of input to output trajectories. The outcome of our learning rule is a synaptic balancing between the incoming and outgoing synapses of every neuron. This synaptic balancing rule is consistent with many known aspects of experimentally observed heterosynaptic plasticity, and moreover makes new experimentally testable predictions relating plasticity at the incoming and outgoing synapses of individual neurons. Overall, this work provides a novel, practical local learning rule that exactly preserves overall network function and, in doing so, provides new conceptual bridges between the disparate worlds of the neurobiology of heterosynaptic plasticity, the engineering of regularized noise-robust networks, and the mathematics of integrable Lax dynamical systems.

## Author summary

The precise pattern of synaptic connections in a neural network entirely determines the network’s function. Learning rules are programs for modifying synapses to allow the network to attain some functional goal. By carefully tweaking synaptic connectivity, neural networks are able learn new tasks, form new memories, and stabilize neural activity. Due to the complexity of network structure, there are many different synaptic weight patterns that can in principle perform the same computation. These different solutions, however, may be more or less desirable in other other ways, such as their vulnerability to unreliable components in the network. In this paper, we present a synaptic learning rule which locates robust solutions among many synaptic weight configurations that equivalently perform the same task. Our biologically plausible rule relies on information which is entirely locally available to each neuron and possesses a number of desirable mathematical properties. Our analysis reveals that networks are most robust when the aggregate incoming and outgoing synapses are balanced in every neuron. Intriguingly, our learning rule makes predictions which resemble widely experimentally observed patterns of compensatory heterosynaptic plasticity in cortical dendrites.

## Introduction

As any neural circuit computes, it is subject to additional fluctuations either within the circuit or from other brain regions [1–4], and these fluctuations can impair performance [5–8]. The fundamental puzzle we address is what kind of plasticity rule can make the dynamics of a neural circuit more robust to such fluctuations in a manner that: (1) works for any task; (2) is completely agnostic to the learning rule used to solve a task; and (3) does not impair network performance after a task is learned. Our main contribution is the discovery of a plasticity rule that provably accomplishes all of three objectives for any nonlinear recurrent neural network with a homogeneous nonlinearity, such as the commonly studied rectified linear function. Furthermore, our plasticity rule is biologically plausible and computable using only information that is locally available at each synapse and its adjacent neurons. Finally, at a mathematical level our plasticity rule connects to the theory of integrable dynamical systems [9–11] and heat diffusion, while experimentally, its features are similar to observed aspects of heterosynaptic plasticity [12–16].

The key idea behind our learning rule is to exploit a many-to-one mapping between patterns of synaptic strength and the task, or the temporal input-output map implemented by a recurrent neural network. Indeed, modern neural network models of animal behavior typically possess a large number of tunable parameters—far more than necessary to perform a given simple behavior. In this overparameterized regime, it has been observed across multiple behaviors and organisms that many distinct model configurations are able to generate equivalent levels of task performance [17, 18]. This observation has spurred theoretical and numerical investigations into specific means by which a task may constrain network connectivity and function [19–23]. However, the complex, nonlinear nature of many classes of network models in neuroscience—notably recurrent neural networks (RNNs)—has made it difficult in many cases to obtain a precise theoretical characterization of the space of synaptic patterns that all map to the same task [24].

Besides a theoretical interest in formally characterizing equivalence classes of synaptic weights that solve the same task, we are also motivated by a complementary biological question: how might neural systems actually implement local plasticity rules which take advantage of network overparameterization to maintain desirable functional properties, including task performance, in the presence of internal and external sources of variation? Studies of homeostatic plasticity in cortical circuits have shed light on synaptic mechanisms thought to maintain stable computation, including synaptic scaling, heterosynaptic plasticity, and other compensatory processes [13, 25–28]. However, a fundamental neuroscientific question remains: can such local homeostatic or compensatory plasticity rules operate in such a way so as not to impair task performance, while still accruing some other additional benefit?

In this work, we provide insights into both the nature of overparameterization in recurrent neural networks and how local plasticity rules might exploit this overparameterization to specifically improve network robustness without impairing task performance. First we precisely characterize the equivalence classes of synaptic weights that all solve the same task in nonlinear recurrent networks with homogenous nonlinearities. Using this characterization, we derive a simple associated plasticity rule that locally modifies synaptic weights while remaining within these equivalence classes. Intriguingly, we find our theoretically derived plasticity rule resembles experimentally observed heterosynaptic plasticity rules.

### Outline of this paper

The structure of this paper is as follows. First, we introduce the nonlinear recurrent network model and a transformation of network weights that exactly preserves task performance. Second, we formalize a notion of noise robustness in recurrent networks and show that this quantity is convex with respect to the coordinates of the task-preserving transformation. We then derive a dynamical transformation of the network, called synaptic balancing, which maximizes robustness while exactly maintaining task performance, and which is implementable entirely by local update rules. We next show that synaptic balancing is exponentially stable in any recurrent network which does not contain irreducible feedforward structure. Subsequently, we introduce several generalizations of our model and show that synaptic balancing is an instance of a broader class of integrable dynamical systems on synaptic weight matrices known as Lax dynamical systems. These dynamical systems lead to isospectral flows on matrices that preserve all eigenvalues of the matrix.

Turning to the behavior of synaptic balancing alongside task-relevant learning dynamics, we then prove that a broad class of regularized networks naturally approaches the equilibrium of our rule throrightugh training. We conversely show empirically that our rule is able to improve the task performance of trained networks in previously unseen noisy regimes.

Finally, we address the role of synaptic balancing as a candidate local plasticity rule for maintaining stable network computation in neural circuits. We present exact and approximate solutions to the trajectory of synaptic balancing, deriving a formal connection between synaptic balancing and heat diffusion in a network. In closing, we draw a connection between synaptic balancing and experimentally observed patterns of heterosynaptic plasticity in cortical synapses.

## Results

### A task-preserving transformation defines a manifold of equivalent recurrent networks

In this section, we first describe a class of nonlinear recurrent neural networks that has been extensively studied in diverse contexts [6, 7, 19, 23, 24, 29–31]. We then describe a natural symmetry acting on the weight space of these neural networks that *exactly preserves* the entire temporal mapping of input to output trajectories. Since the input-output mapping determines the task a neural network performs, the action of this symmetry enables us to traverse the manifold of weight configurations that preserve the task performed by any neural network.

#### Recurrent network model

Consider a recurrent rate network of *N* neurons with an *N* × *N* synaptic weight matrix **J**, such that neuron *j* is connected to neuron *i* with synaptic weight *J*_*ij*_. The vector of neural activity $x\in R^{N}$ and readout vector **y** evolve under a time-varying external input **u** as

$$\begin{array}{c} \tau \dot{x}=f_{W}\left(x,u\right)=-x+J\phi \left(x\right)+W^{\text{in}}u, \end{array}$$ (1)

$$\begin{array}{c} y=W^{\text{out}}x. \end{array}$$ (2)

Here *τ* is a fast timescale of neural dynamics, *ϕ* is a typically nonlinear scalar function applied element-wise to **x**, and the tuple $W=(W^{\text{in}},\;J,\;W^{\text{out}})$ is the weight configuration which parameterizes the network.

We assume for simplicity that neural activity is initialized at the origin and runs until some time *T*. The dynamics of the full network, and in particular the output trajectory **y**(*t*), are a deterministic function of the input trajectory **u**(*t*) and the weight configuration W. More formally, the input-output map *F*_RNN_ computed by the network is the map from input to output trajectories under a particular weight configuration:

$$\begin{array}{c} F_{\text{RNN}}\left(W\right):\;{\left\{u\left(t\right)\right\}}_{t=0}^{T}\mapsto {\left\{y\left(t\right)\right\}}_{t=0}^{T} \end{array}$$ (3)

In this work, we introduce a model of synaptic updates whose properties are well behaved when *ϕ* is a homogeneous function; that is, when *ϕ*(*αx*) = *αϕ*(*x*) for all x∈R and $\alpha \in R^{+}$. Common activation functions which satisfy this condition include the linear (*ϕ*(*x*) = *x*) and rectified linear (*ϕ*(*x*) = max(0, *x*)) units. In the main text of this paper we assume that *ϕ* is homogeneous, however, in S1 Appendix. 4 we give an extension to the more general class of positively homogeneous nonlinearities, which satisfy *ϕ*(*sx*) = *s*^*k*^*ϕ*(*x*), for any *s* > 0 and k∈R.

#### Task-preserving transformation

We now describe a symmetry in weight space that exactly preserves the map (3). Indeed, the observation that many distinct weight configurations may yield quantitatively similar network behavior is widespread both in neuroscience, where simulations of the crustacean stomatogastric ganglion found a range of synaptic strengths producing a given network output [17], and in machine learning, in which non-convex cost functions possess many roughly equivalent local minima [19, 32]. In the class of networks we study, part of the degeneracy between weights and a given task originates from a symmetry in weight space that exactly preserves the deterministic input-output map computed by the network. Intuitively, this symmetry scales neurons’ inputs while reciprocally scaling their outputs, such that the overall function computed by each neuron remains intact.

More precisely, consider a map *π*, parameterzied by $h\in R^{N}$, which takes as input a weight configuration $W=(W^{\text{in}},\;J,\;W^{\text{out}})$, and produces as an output the weight configuration

$$\begin{array}{c} \pi_{h}\left(W\right)=\left(e^{-H}W^{\text{in}},\;e^{-H}Je^{H},\;W^{\text{out}}e^{H}\right). \end{array}$$ (4)

Here **H** denotes the diagonal matrix diag{**h**}.

It is worth emphasizing some basic properties of the transformation *π*_**h**_. The sign of synapses is preserved, and synapses which are initially zero remain so. Thus, the transformation does not modify the basic wiring diagram of the network—for example by creating or removing synapses, or changing excitatory to inhibitory synapses and vice versa. Rather, it scales the strength of existing synapses by a positive quantity. In addition *π*_**h**_ acts on the recurrent weight matrix **J** through a similarity transformation. Thus the entire eigenspectrum of the recurrent weight matrix is conserved under this map.

However, *π*_**h**_ satisfies an additional important condition: it exactly preserves the entire input-output output map of the network. For this reason, we refer to *π*_**h**_ as the task-preserving transformation. More formally, for any (finite) value of **h**,

$$\begin{array}{c} F_{\text{RNN}}\left(W\right)=F_{\text{RNN}}\left(\pi_{h}\left(W\right)\right). \end{array}$$ (5)

**Fig 1:**
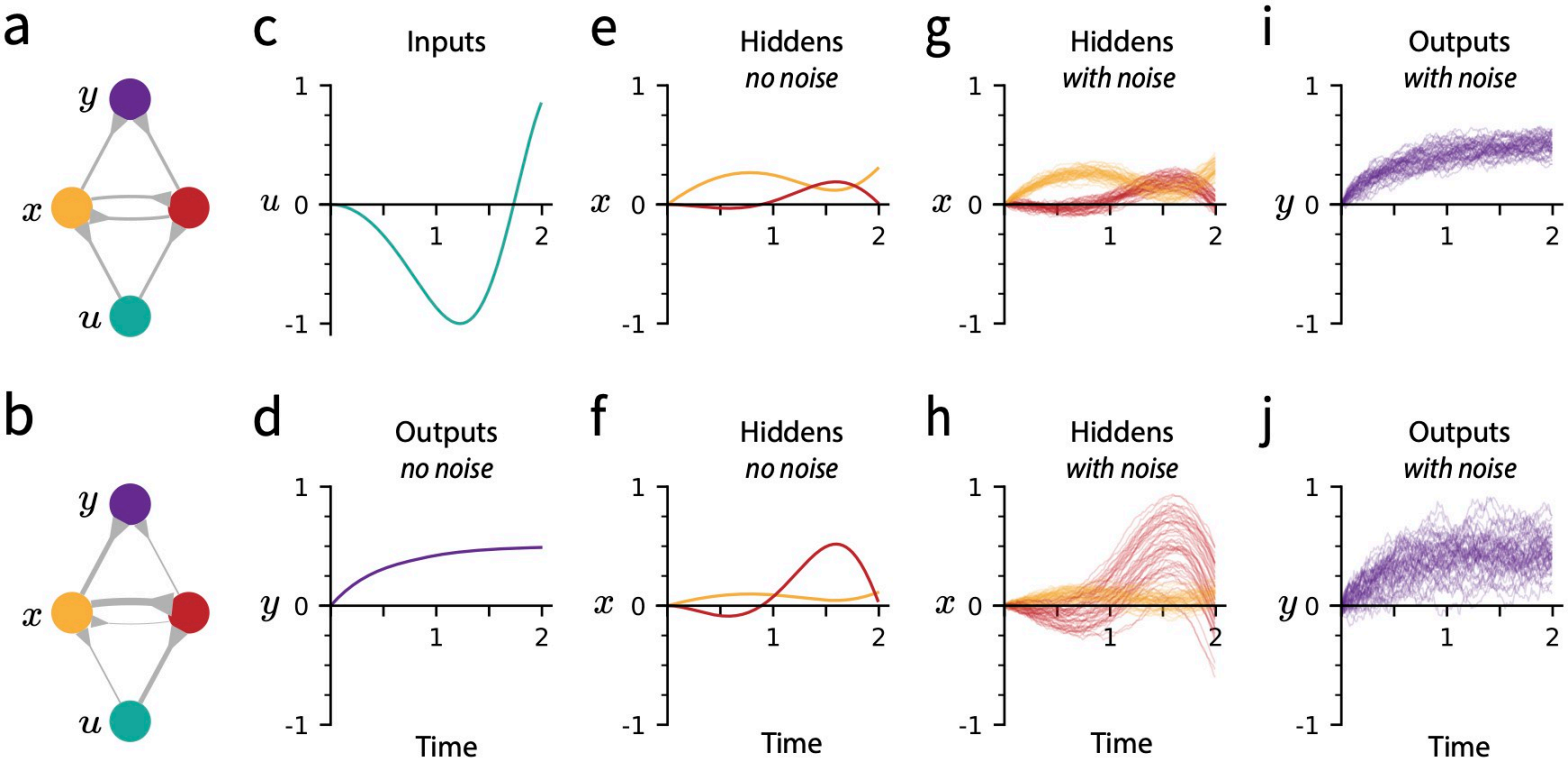
Two nonlinear recurrent networks consisting of input, hidden, and output neurons possess an identical input-output relationship, but behave differently in the presence of noise. **(a-b)** A single input *u* (teal) drives two recurrent neurons *x*_1_ (yellow) and *x*_2_ (red) which project to a single output *y* (purple). The connectivity patterns of the two networks are related by the task-preserving transformation (4). Line thickness denotes synaptic strength. **(c)** Input trajectory, fed to both networks. Horizontal axis in all panels is time. **(d)** Output trajectory, produced by both networks when run according to the deterministic dynamics (1). **(e-f)** Hidden unit neural activity under deterministic dynamics, for networks **a** and **b** respectively. Trajectories are identical up to a per-neuron scale factor, determined by the parameters of the task-preserving transformation. **(g-h)** Hidden neuron neural activity for networks **a** and **b** respectively when additive Gaussian noise is injected into the neural dynamics (1). 50 trials are shown. **(i-j)** Output neuron neural activity in the noisy case.

This claim is summarized in Fig 1, which shows in panels a—f that while the dynamics of the hidden units for two networks related by the task-preserving transformation are different, the output trajectories agree. Eq (5) is proved in the following proposition.

**Proposition 1** (Task-preserving transformation). *The transformation* (4) *exactly preserves the input-output relationship of the neural dynamics* (1). *Given two networks receiving the same time course of inputs* **u**(*t*) *and with weight configurations* $W^{0}$ *and* $W=\pi_{h}\left(W^{0}\right)$ *respectively, then*:

1. *If* **x**^0^(*t*) *is the time course of hidden unit neural activity under* $W^{0}$, *then* *e*^−**H**^**x**^0^(*t*) *is the time course of hidden unit neural activity under* W.
2. *If* **y**^0^(*t*) *is the time course of output neural activity under* $W^{0}$, then **y**^0^(*t*) *is also the time course of output unit neural activity under* W.

The proof of proposition 1 is straightforward and can be found in S1 Appendix 1.

Given an initial weight configuration $W^{0}$, we define the *task-preserving manifold* of $W^{0}$ to be the manifold of weight configurations accessible from $W^{0}$ by the task-preserving transformation, i.e., the orbit of $W^{0}$ under the symmetry *π*_**h**_: $\left\{\pi_{h}\left(W^{0}\right):\;h\in R^{N}\right\}$. The vector **h** provides a set of coordinates on this manifold. When referring to the task-preserving manifold we implicitly assume there exists some reference weight configuration $W^{0}$ at which **h** = 0.

### A measure of robustness to noise in neural activity

We have shown that every weight configuration on the task-preserving manifold computes the same deterministic input-output map. However, biological systems are inherently noisy [1], and it has been found that cortical spike trains vary at the sub-millisecond level across trials with identical inputs [2–4]. Interestingly, even two networks which compute the same deterministic input-output map may exhibit differing responses to neural noise. In Fig 1, we give an example of two networks which perform identically in the deterministic setting but which respond differently when noise is injected to hidden units.

In this section we introduce a quantitative notion of a network’s robustness to noise, which we call sensitivity. This function describes the degree to which noise in neural activity may interfere with the underlying computation of the network. We connect our notion of sensitivity to the gain of neurons in the network and show that the sensitivity is well-behaved on the task-preserving manifold; in particular, that it possesses a convex geometry in the coordinates **h**.

#### Robustness of neural dynamics to random perturbations

A recurrent network whose dynamics are easily perturbed by small variations in neural activity is unlikely to perform a task robustly in the presence of neural noise. To capture this notion, we consider the magnitude of the response to a small, isotropic Gaussian perturbation $\delta ∼N(0,\varepsilon^{2}I)$ to the neural dynamics (1), averaged over the distribution of states X visited by the network during a task:

$$\begin{array}{c} \frac{1}{\varepsilon^{2}}{\left\langle \|f\left(x+\delta ,u\right)-f\left(x,u\right)\right\|}_{2}^{2}\rangle_{\delta ,x}, \end{array}$$

where for simplicity we have dropped from **f** an explicit dependence on the weights W. We define the sensitivity of a network to be the first-order approximation of this quantity,

$$\begin{array}{c} S={\left\langle {\left\|\frac{\partial f}{\partial x}\right\|}_{F}^{2}\right\rangle }_{x∼X}, \end{array}$$ (6)

where the approximation is made via Taylor expansion of **f** about **x**. Networks with lower sensitivity are less easily pushed away from their original trajectories and are therefore more likely to be robust to noise while performing tasks, as is illustrated in Fig 1(g)–1(j). In this paper, robustness refers to *S*^−1^, the reciprocal of sensitivity.

In the case of the neural dynamics (1), the Jacobian matrix takes the form,

$$\begin{array}{c} \frac{\partial f}{\partial x}=-I+J\;\text{diag}\{\phi^{\prime }\left(x\right)\}. \end{array}$$ (7)

This expression makes use of the gain, *ϕ*′(*x*_*i*_), of neuron *i*. With respect to the distribution of neural activity X, the gain has first and second moments

$$\begin{array}{c} \begin{array}{cc} \mu_{i} & ={\left\langle \phi^{\prime }\left(x_{i}\right)\right\rangle }_{x_{i}∼X_{i}}\\ \sigma_{i}^{2} & ={\left\langle \phi^{\prime }{\left(x_{i}\right)}^{2}\right\rangle }_{x_{i}∼X_{i}} \end{array} \end{array}$$ (8)

for each neuron *i*. We may rewrite the definition of sensitivity (6) using the Jacobian of the neural dynamics (7) in terms of the moments (8) to obtain

$$\begin{array}{c} \begin{array}{cc} S & ={\left\langle {\left\|\frac{\partial f}{\partial x}\right\|}_{F}^{2}\right\rangle }_{x∼X}\\ & ={\left\langle \text{Tr}[(-I+J\;\text{diag}\left\{\phi^{\prime }\left(x\right)\right)(-I+J\;\text{diag}{\left\{\phi^{\prime }\left(x\right)\right)}^{T}]\right\rangle }_{x∼X}\\ & =\text{Tr}[I-J\;{\left\langle \text{diag}\{\phi^{\prime }\left(x\right)\}\right\rangle }_{X}-{\left\langle \text{diag}\{\phi^{\prime }\left(x\right)\}\right\rangle }_{X}\;J^{T}+J{\left\langle \text{diag}{\left\{\phi^{\prime }\left(x\right)\right\}}^{2}\right\rangle }_{X}J^{T}]\\ & =N-2\;\text{Tr}[J\;{\left\langle \text{diag}\{\phi^{\prime }\left(x\right)\}\right\rangle }_{X}]+\text{Tr}[J{\left\langle \text{diag}{\left\{\phi^{\prime }\left(x\right)\right\}}^{2}\right\rangle }_{X}J^{T}] \end{array} \end{array}$$

$$\begin{array}{c} S=\sum_{ij}\sigma_{j}^{2}J_{ij}^{2}-2\sum_{i}\mu_{i}J_{ii}+N. \end{array}$$ (9)

The sensitivity, then, can be succinctly written as a function of the recurrent weights and the moments of the neural gains. However, because the moments *μ* and *σ*^2^ are defined as an average over the distribution of network states that depends—generally intractably—on the weights themselves, sensitivity is not, as it may appear, a straightforward quadratic function of **J**. We next show that, perhaps surprisingly, sensitivity turns out to be well behaved as a function on the task-preserving manifold.

#### Sensitivity is a convex function on the task-preserving manifold

Because networks attaining weight configurations of lower sensitivity might see improved task performance in noisy environments (as in Fig 1), it is useful to analytically characterize how *S* varies as a function of network weights. We have just mentioned the complications of studying this question in general. Here, we find that *S* becomes tractable when we consider its variation on the task-preserving manifold, parameterized by the coordinates **h**. In particular, we show that *S* is convex with respect to **h**.

**Proposition 2**. *When considered as a function on the coordinates* **h** *of the task-preserving manifold, the sensitivity S is a convex function*

$$\begin{array}{c} S=\sum_{ij}c_{ij}^{0}e^{p(h_{j}-h_{i})}+S_{\text{const}} \end{array}$$ (10)

*for some constants* ${\left\{c_{ij}^{0}\right\}}_{i,j=1}^{N}$, *p*, *and* *S*_const_, *where* $c_{ij}^{0}\geq 0$ *for all i*, *j*.

*Proof*. Assume there is some original network $W^{0}=\left(W^{\text{in},0},J^{0},W^{\text{out},0}\right)$, whose distribution of neural activity is $X^{0}$, and whose gains have moments ${\left\{\mu_{i}^{0}\right\}}_{i=1}^{N}$ and ${\left\{{\left(\sigma_{i}^{0}\right)}^{2}\right\}}_{i=1}^{N}$. Consider a transformed network $W=\pi_{h}\left(W^{0}\right)$, with analogous quantities X, ${\left\{\mu_{i}\right\}}_{i=1}^{N}$, and ${\left\{\sigma_{i}^{2}\right\}}_{i=1}^{N}$. From (4), the recurrent connectivity of the transformed network is $J_{ij}=J_{ij}^{0}e^{h_{j}-h_{i}}$. The sensitivity of the transformed network in terms of the task-preserving transformation is obtained by plugging this *J*_*ij*_ into (9):

$$\begin{array}{c} S=\sum_{ij}\sigma_{j}^{2}{\left(J_{ij}^{0}\right)}^{2}e^{2(h_{j}-h_{i})}-2\sum_{i}\mu_{i}J_{ii}^{0}+N. \end{array}$$ (11)

For convexity, it is sufficient to show that the moments of the gain, *μ*_*i*_ and $\sigma_{i}^{2}$, are constant with respect to the task-preserving transformation; i.e., that $\mu_{i}=\mu_{i}^{0}$ and $\sigma_{i}^{2}={\left(\sigma_{i}^{0}\right)}^{2}$ for each neuron *i*. From Prop. 1, the transformed neural activity can be written in terms of the original rates as $x_{i}=e^{-h_{i}}x_{i}^{0}$ for all *t* > 0. By assumption, *ϕ* satisfies *ϕ*(*αx*) = *αϕ*(*x*), and therefore *ϕ*′(*αx*) = *ϕ*′(*x*), for all x∈R and $\alpha \in R^{+}$. So $\phi^{\prime }\left(x_{i}\right)=\phi^{\prime }\left(e^{-h_{i}}x_{i}^{0}\right)=\phi^{\prime }\left(x_{i}^{0}\right)$, and

$$\begin{array}{c} \mu_{i}=\left\langle \phi^{\prime }\left(x_{i}\right)\right\rangle =\left\langle \phi^{\prime }\left(x_{i}^{0}\right)\right\rangle =\mu_{i}^{0}\;\\ \sigma_{i}^{2}=\left\langle \phi^{\prime }{\left(x_{i}\right)}^{2}\right\rangle =\left\langle \phi^{\prime }{\left(x_{i}^{0}\right)}^{2}\right\rangle ={\left(\sigma_{i}^{0}\right)}^{2} \end{array}$$

for each neuron *i*. Therefore the moments of the gains are constant with respect to **h**.

We conclude that (11) takes the form of (10), where the constants are $c_{ij}^{0}={\left(\sigma_{j}^{0}\right)}^{2}{\left|J_{ij}^{0}\right|}^{2}\geq 0$, *p* = 2, and $S_{\text{const}}=-2\sum_{i}\mu_{i}^{0}J_{ii}^{0}+N$. As (10) is a positive linear combination, with constant coefficients, of exponentiated linear functions of **h** (which are convex), then it is in turn a convex function of **h** [33, ch. 3].

#### Other notions of robustness

Our definition of sensitivity (6) is chosen to emphasize the aspect of recurrent networks which is typically most crucial to task performance; that is, the neural dynamics. However, there are other notions of robustness which are useful to mention. Here we briefly address how the task-preserving transformation (4), parameterized by **h**, interacts with several additional relevant notions of sensitivity.

First, consider the sensitivity *S*^**u**→**y**^ of the output trajectory {**y**(*t*)} to fluctuations in the input trajectory {**u**(*t*)}. In our analysis, no choice of **h** can affect this quantity, precisely because the task-preserving transformation exactly preserves the input-output map. Put differently, this notion of sensitivity is intrinsic to the function being computed, not to the manner in which the recurrent network computes it.

Second, consider the sensitivity *S*^**x**→**y**^ of the output trajectory {**y**(*t*)} to fluctuations in the time course of neural activity {**x**(*t*)} (Fig 1(i) and 1(j)), and respectively, the sensitivity *S*^**u**→**x**^ of neural activity {**x**(*t*)} to fluctuations in the input trajectory {**u**(*t*)}. These sensitivities can be individually minimized by taking **h** → −∞ to minimize the norm of **W**^out^ and, respectively, **h** → ∞ to minimize the norm of **W**^in^. The resulting tradeoff between *S*^**x**→**y**^ and *S*^**u**→**x**^ is separate from the problem of minimizing our choice of *S*, and it may be solved independently of the method we now present. In what follows, we will present a local learning rule that maximizes the robustness of the neural dynamics. We will further show that these dynamics will never result in the case of **h** or any of its elements becoming ±∞.

### A local learning rule maximizes robustness while preserving task performance

We have introduced the task-preserving manifold as the set of weight configurations accessible by a symmetry transformation which preserves a given deterministic input-output map. We have also shown that the sensitivity of neural dynamics to small perturbations in activity—the generally intractable quantity *S*—is well behaved when constrained to the task-preserving manifold. A simple question emerges: how might networks traverse the task-preserving manifold to maximize robustness while preserving underlying task performance? To address this question, we derive gradient descent dynamics that maximize network robustness in the coordinates of the task-preserving manifold. We find, perhaps surprisingly, that these dynamics are wholly implementable by biologically plausible local computations within neurons and synapses.

From (10), the problem of maximizing robustness *S*^−1^ is equivalent to that of minimizing the total cost:

$$\begin{array}{c} C=\sum_{ij}c_{ij}, \end{array}$$ (12)

where *c*_*ij*_ is the synaptic cost

$$\begin{array}{c} c_{ij}=\sigma_{j}^{2}{\left|J_{ij}\right|}^{p} \end{array}$$ (13)

$$\begin{array}{cc} & =c_{ij}^{0}e^{p(h_{j}-h_{i})}, \end{array}$$ (14)

with *p* = 2, and in the second line we have rewritten the synaptic costs in terms of the initial cost $c_{ij}^{0}={\left(\sigma_{i}^{0}\right)}^{2}{\left|J_{ij}^{0}\right|}^{p}$ and the coordinates **h**, obtained by writing out (13) in terms of the task-preserving transformation $J_{ij}=J_{ij}^{0}e^{h_{j}-h_{i}}$ (4).

We call the network connected if the directed graph whose edge weights are given by the initial synaptic cost matrix **C**^0^ is connected. We assume, without loss of generality, that the network is connected (if not, a similar theory applies to each connected component separately).

We turn to deriving synaptic dynamics that transforms **h** over time so as to maximize robustness on the task-preserving manifold. Until now we have treated the vector **h** as a free coordinate on the task-preserving manifold. Henceforth we consider it as a function of time, initializing at the origin and evolving under gradient descent on the total cost. Similarly, we now view $W=\left(W^{\text{in}},J,W^{\text{out}}\right)=\pi_{h}\left(W^{0}\right)$ as a time-varying weight configuration, with initial value $W^{0}$ corresponding to **h** = 0.

Let the time derivative of **h** be denoted by $\dot{h}=g$, which we refer to as the neural gradient vector. The dynamics of gradient descent on *C* with respect to **h** is given by

$$\begin{array}{c} g=-\gamma \frac{\partial C}{\partial h}, \end{array}$$ (15)

where γ∼O(1) is the descent rate. We assume a separation of timescales, such that neural dynamics (1) are much faster than the weight dynamics (15).

The synaptic costs (13) obey

$$\begin{array}{c} \frac{\partial c_{ij}}{\partial h_{k}}=p\;c_{ij}\;\left(\delta_{kj}-\delta_{ki}\right), \end{array}$$ (16)

where *δ* is the Kronecker delta. In the present regime where γ∼O(1) and p∼O(1), we are free to adopt throughout *γp* = 1 for notational convenience. Using this, we evaluate the right hand side of (15) via (12) and (16) to find,

$$\begin{array}{c} g_{k}=\sum_{j}c_{kj}-\sum_{i}c_{ik}. \end{array}$$ (17)

Finally, to see how synaptic strengths are updated, we differentiate the task-preserving transformation $J_{ij}=J_{ij}^{0}e^{h_{j}-h_{i}}$ with respect to time, finding that

$$\begin{array}{c} {\dot{J}}_{ij}=J_{ij}\left(g_{j}-g_{i}\right), \end{array}$$ (18)

$$\begin{array}{c} {\dot{W}}_{ik}^{\text{in}}=-W_{ik}^{\text{in}}g_{i} \end{array}$$ (19)

$$\begin{array}{c} {\dot{W}}_{kj}^{\text{out}}=W_{kj}^{\text{out}}g_{j} \end{array}$$ (20)

Eqs (17) through (20), along with the definition of synaptic costs (13), are self-contained and collectively comprise the dynamics of our proposed update rule, which we call synaptic balancing.

Interestingly, we find that synaptic balancing is entirely implementable by local computations in a network. First, costs (13) are computed at the synapse as a product of the square of the synaptic weight and the presynaptic average gain. Second, neural gradients (17) are computed centrally in the neuron by aggregating and comparing the costs of incoming and outgoing synapses. Third, synaptic weights (18) are updated in proportion to the difference between the presynaptic and postsynaptic neural gradients.

**Fig 2:**
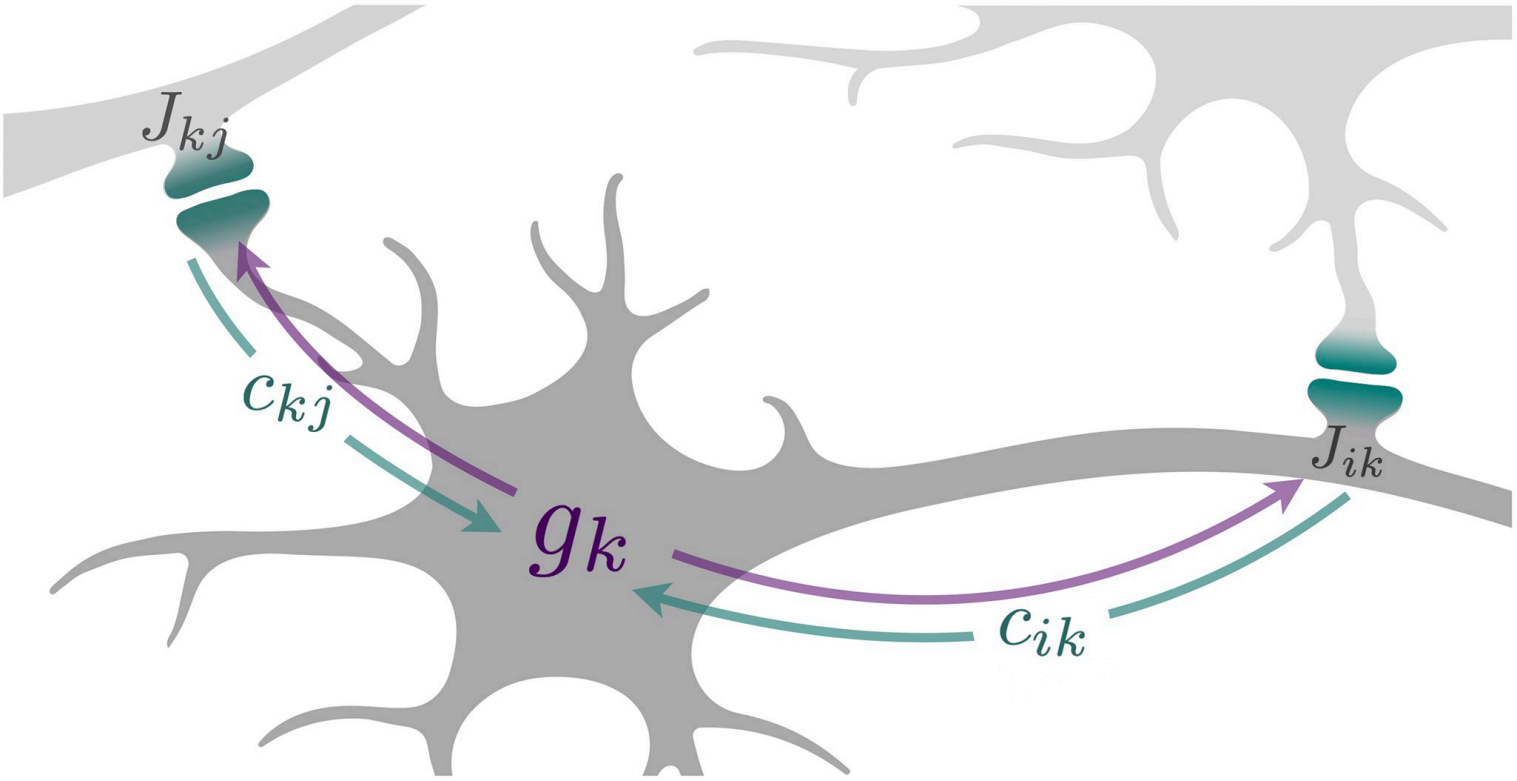
The local computations underlying synaptic balancing. Each synapse (indicated in teal) calculates a cost as a function of synaptic strength, as in (13). Neuron *k* receives signals of incoming synaptic cost *c*_*kj*_ and outgoing synaptic cost *c*_*ik*_ (teal arrows from synapses to soma) and computes the difference *g*_*k*_ as in (17). The signal *g*_*k*_ then propagates outwards (purple arrows from soma to synapses) to modify the strength of incoming and outgoing synaptic connections, as in (18), such that the total incoming and total outgoing costs are eventually balanced in every neuron.

In this scheme we assume that the synaptic weights, synaptic costs, and neural gradients can be represented as biophysical quantities inside neurons and synapses. We suppose, as depicted in Fig 2, that in one direction a neuron traffics its gradient from the soma to incoming and outgoing synapses, and, in the other, it traffics synaptic costs from synapses to the soma. Finally, we assume that each synapse is able to measure and store certain statistics of presynaptic activity, namely, the average presynaptic gain. This assumption is in line with other models of synaptic plasticity which introduce some form of leaky integration of neural activity, e.g. the sliding threshold of the BCM rule [34, p. 288].

Notably, although the coordinates **h** and the task-preserving transformation *π*_**h**_ are of instrumental value in deriving and analyzing our rule, the biological procedure just described implements synaptic balancing without an explicit representation of **h**.

Next, we study the equilibrium state of synaptic balancing and offer a simple condition under which an equilibrium exists, thereby guaranteeing the stability of our rule.

### Strongly connected networks attain balanced equilibrium

Synaptic balancing asymptotically converges to a global minimum on the task-preserving manifold, by construction, because it is gradient descent on the convex function *C*. However, a global minimum may not exist at any finite value of the task-preserving manifold coordinates **h**. For example, the convex function *f*(*h*) = *e*^*h*^ on the real line possesses no global minimum at finite *h*, and the asymptotic convergence of gradient descent is not assured. It is therefore important to establish the criteria under which we expect synaptic balancing to stably converge to a (finite) minimizer h*. To do this, we provide two simple criteria: first, a balance condition which describes the set of equilibria of synaptic balancing, and second, a strongly-connected condition which implies the existence of an equilibrium on the task-preserving manifold that attains exponential convergence.

A weight configuration globally minimizes the total cost on the task-preserving manifold if and only if it satisfies the balance condition

$$\begin{array}{c} \sum_{i}c_{ik}=\sum_{j}c_{kj} \end{array}$$ (21)

for each neuron *k*. To see this, observe that because the total cost *C* is convex and differentiable as a function of **h**, a coordinate h* achieves a global minimum if and only if $\frac{\partial C}{\partial h}\left(h^{*}\right)=0$, which, from (15), holds if and only if **g** = 0. Solving for **g** = 0 in (17) yields the balance condition (21).

It is because of (21) that we use the term balancing to describe our rule: the total cost is minimized, and a weight configuration is a stable fixed point of the weight dynamics (18), if and only if the total synaptic costs of each neuron’s incoming and outgoing synapses are equal. It remains unclear, however, whether the balance condition (21) is attainable in every case, or put differently, whether synaptic balancing is stable from every initial condition.

We now state a simple topological criterion which (we will show) implies the stability of synaptic balancing. A recurrent network is said to be strongly connected if between every pair of neurons *i* and *j* there exists a path of synapses, each with positive synaptic cost, from *i* to *j*. Conceptually, this condition simply means that the recurrent connectivity does not possess any embedded directed acyclic structure, such as purely feedforward connections.

With regard to biology, it is of course generally challenging to show that any given biological network is strongly connected. With that said, we believe that the highly recurrent nature of the central nervous system suggests that this assumption may not be far from reality in certain cortical regions. Long-range feedback projections as well as local recurrence in cortex suggest that non-negligible sub-networks of neurons participating in, for example, the ventral visual stream are indeed strongly connected [35, 36]. Such networks are strong candidates for synaptic balancing.

Mathematically, it is known that the connected components of any balanced matrix, i.e., a matrix satisfying (21), are strongly connected [37], and that, conversely, such matrices may be made balanced through a positive diagonal similarity transformation [38].

Before formally describing the stability of our rule, we make the preliminary observation that the synaptic costs in (14) are invariant under the transformation **h** ↦ **h** + *b***1**, for b∈R. Therefore the gradient of the total cost *C* is perpendicular to the all ones vector **1**, and gradient descent on *C* does not explore that direction: From (17), $1^{T}\dot{h}=\sum_{i}g_{i}=0$. Assuming as before that **h**^0^ = 0, synaptic balancing exclusively yields solutions that satisfy

$$\begin{array}{c} 1^{T}h=1^{T}h^{0}=0. \end{array}$$ (22)

**Fig 3:**
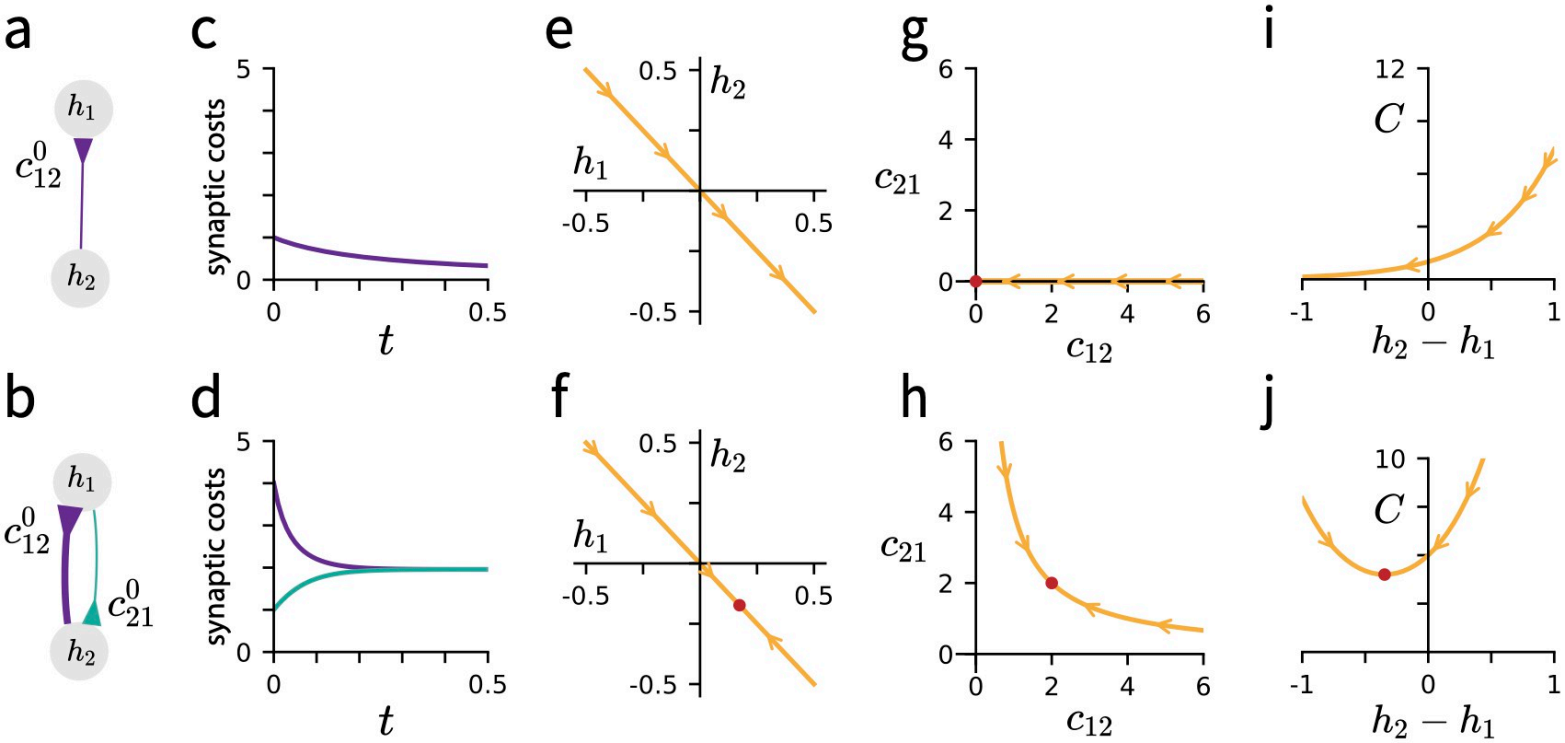
Network topology determines the geometry of the task-preserving manifold and the dynamics of synaptic balancing. Top row: ReLu network with two hidden units connected by a single synapse, corresponding to initial synaptic cost $c_{12}^{0}$. Bottom row: ReLu network network with two hidden units connected reciprocally, with initial synaptic costs $c_{12}^{0}$ and $c_{21}^{0}$. **(a-b)** Network diagrams showing topology and initial synaptic costs, indicated by line thickness. Input and output neurons are not shown. **(c-d)** Trajectory of synaptic costs over the course of synaptic balancing. Line colors match synapse colors in panels (a-b). Panel (c) matches (31) and panel (d) matches (29). **(e-f)** The feasible set of **h** satisfying (22), with flow lines indicating trajectory of synaptic balancing. Panel (f): Red point indicates the (finite) minimizer of the total cost. **(g-h)** Tradeoff between *c*_12_ and *c*_21_ as a function of *h*_2_ − *h*_1_, with red point indicating the optimal value of total cost *C* = *c*_12_ + *c*_21_. **(i-j)** Total cost *C* as a function of *h*_2_ − *h*_1_. (i) Optimal cost is zero, attained at an infinite value of **h**. (j) Optimal cost is positive, attained at finite **h**.

We may now connect these remarks to our weight dynamics, showing that synaptic balancing converges exponentially to a balanced configuration whenever the initial cost matrix is strongly connected. Fig 3 illustrates this point in two-neuron networks, showing the time course of synaptic balancing and the geometry of the cost function. In the first case of a single feedforward connection, a stable equilibrium is not reached at any finite **h**. In the second case of recurrent connections but asymmetric initial synaptic costs, the network converges to a stable equilibrium at finite **h** and positive total cost *C*, with symmetric final costs. A short proof is provided in the following proposition.

**Proposition 3**. *In a strongly connected network, synaptic balancing converges to a globally exponentially stable equilibrium, which is the unique solution to* (22) *and* (21) *on the task-preserving manifold*.

*Proof (Sketch)*. Because synaptic balancing does not increase the total cost *C* = ∑_*ij*_ *c*_*ij*_ (12), and because the synaptic costs are nonnegative, we have that *c*_*ij*_ ≤ *C*^0^ along the trajectory of synaptic balancing for all *i*, *j*. If $c_{ij}^{0}>0$, then since $c_{ij}=c_{ij}^{0}e^{p(h_{j}-h_{i})}$ (14), it follows directly that $e^{h_{j}-h_{i}}\leq {\left(C^{0}/c_{ij}^{0}\right)}^{1/p}<\infty$. If $c_{ij}^{0}=0$, then an “indirect” upper bound for $e^{h_{j}-h_{i}}$ is obtained by collapsing the direct upper bounds along a path of positive synaptic costs from *j* to *i*: In the case of a single intervening neuron *k*, for example, $e^{h_{j}-h_{i}}=e^{h_{j}-h_{k}}e^{h_{k}-h_{i}}\leq {\left(C^{0}/c_{kj}^{0}\right)}^{1/p}{\left(C^{0}/c_{ik}^{0}\right)}^{1/p}$. By the strong connectedness assumption, such a path exists between every *i* and *j*, so we may in this way obtain upper bounds on *h*_*j*_ − *h*_*i*_, and therefore on |*h*_*j*_ − *h*_*i*_|, for all *i*, *j*. Combined with the constraint ∑_*i*_ *h*_*i*_ = 0 (22), it follows that **h** is contained in a compact set over the trajectory of synaptic balancing. We conclude that the minimizer of *C*, a convex function of **h**, is attained in this set. To show that the minimizer is unique and globally exponentially stable, it is sufficient to show that under strong connectedness, *C* is strongly convex on the sublevel set of *C*^0^. This is shown in S1 Appendix 2 with a more detailed proof.

In summary, synaptic balancing consists of local synaptic updates which are stable in any recurrent network that does not possess directed acyclic structure. By minimizing a convex cost on the task-preserving manifold, the rule maximizes the robustness of neural dynamics to noise while maintaining underlying task performance. In the following section we will mention a few useful generalizations of our model.

### Generalizations and a connection to Lax dynamical systems

We have derived synaptic balancing as gradient descent on a behaviorally-relevant convex function—the sensitivity of the network to neural noise—and characterized the stability and equilibria of the rule. We now observe that our framework for synaptic balancing admits several generalizations: first, a broader class of synaptic costs, of which sensitivity is a particularly salient example, and second, a yet more general class of matrix-valued dynamical systems known as Lax dynamics, which have found widespread applications in fields from physics [9] to optimization and numerical linear algebra, where they are known as isospectral flows [10, 11].

#### A general class of well-behaved synaptic costs

The expression for synaptic costs (13) was derived to maximize robustness by minimizing the behaviorally relevant sensitivity function (6). Generally, however, the framework presented here admits a broad class of synaptic cost functions. If the total cost is the sum of synaptic costs, as in (12), then the synaptic cost *c*_*ij*_ may be an arbitrary fixed function of *J*_*ij*_. Importantly, the stability of the resulting weight dynamics, and the optimality of any equilibria, are not guaranteed in general.

A sensible class of synaptic costs which are convex in **h** are the power-law costs

$$\begin{array}{c} c_{ij}=\alpha_{ij}{\left|J_{ij}\right|}^{p}, \end{array}$$ (23)

where *α*_*ij*_ ≥ 0 for all *i*, *j*, and *p* > 0. This definition includes sensitivity (13) as a special case, with $\alpha_{ij}=\sigma_{j}^{2}$ and *p* = 2. Other costs of the power-law form include the *ℓ*_1_ and *ℓ*_2_ matrix penalties studied in statistics and machine learning [39, Ch. 3.4]. Notably, the power-law costs (23): *i)* obey (14), *ii)* correspond to neural gradients of the form (17), and *iii)* inherit the stability result of Prop. 3, extending the findings of this work to a broader class of cost function. Robustness, specifically, is only maximized with the particular choice of costs (13).

#### Synaptic Lax dynamics

A yet further generalization of our synaptic update framework dispenses with a cost function entirely. A Lax dynamical system is an evolution on an *N* × *N* matrix **A** such that

$$\begin{array}{c} \dot{A}=\left[A,\;k\left(A\right)\right];\;A\left(0\right)=A_{0}, \end{array}$$ (24)

where $k:R^{N\times N}\to R^{N\times N}$ is a matrix-valued function which depends on time only through its argument **A**, and [⋅, ⋅] denotes the Lie bracket, which is defined as

$$\begin{array}{c} \left[A,\;B\right]=AB-BA. \end{array}$$

An important property of Lax dynamics is that the evolution of **A** takes the form of a time-varying similarity transformation. To see this, consider the *N* × *N* matrix **K** which evolves in time according to

$$\begin{array}{c} \dot{K}=Kk\left(A\right);\;K\left(0\right)=I. \end{array}$$

It is straightforward to show that **K**(*t*) is nonsingular for all *t* and that the similarity transformation

$$\begin{array}{c} A\left(t\right)=K{\left(t\right)}^{-1}A_{0}K\left(t\right) \end{array}$$

is the unique solution of (24). As a consequence, Lax dynamical systems conserve the entire spectrum of the matrix **A** and are sometimes referred to as isospectral flows.

Synaptic balancing is a specific instance of Lax dynamics. We may rewrite (18) as

$$\begin{array}{c} \dot{J}=\left[J,\;\text{diag}\left\{g\right\}\right], \end{array}$$ (25)

noting that the elements of **g** depend on time only through a dependence on the elements of **J**. Thus this dynamics is a special case of the general definition (24).

The Lax dynamics of synaptic balancing (25) encompass a very general form of task-preserving local learning rule. If the neural gradient *g*_*k*_ is allowed to depend arbitrarily on the incoming and outgoing weights of neuron *k*, then synaptic Lax dynamics remains confined to the task-preserving manifold and is fully locally computable in the sense of Fig 2. Further, all quantities which are conserved on the task-preserving manifold—including the spectrum of the recurrent weight matrix and the product of synaptic weights along every directed closed loop—are conserved by the synaptic Lax dynamics as well.

Synaptic Lax dynamics, if chosen in such a way to be stable, might be used to regulate any number of structural properties of the network. Any equilibrium of the synaptic Lax dynamics satisfies the equality condition *g*_*i*_ = *g*_*j*_ for all pairs (*i*, *j*), which generalizes the balance condition (21). For choices of neural gradient for which this equilibrium is attainable and stable, the synaptic Lax dynamics offer a flexible framework for adapting network weights without affecting task performance. For example, [40] studies the problem of stably balancing the maximum incoming and outgoing synaptic strengths, and [41] studies the problem of balancing the incoming and outgoing products of synapses. Each of these aims could be implemented by the synaptic Lax dynamics through proper choice of *g*_*k*_. Our work unifies these efforts under a general framework of continuous-time weight dynamics (25), and, unlike previous approaches, derives from functional considerations a particular form of neural gradient that provably minimizes a behaviorally-relevant cost function on the task-preserving manifold.

### Regularized networks are nearly balanced

Our focus so far has been on establishing theoretical links between the balance condition, noise-robust computation and the proposed local plasticity rule. We now turn to studying the interaction of these concepts with other forms of plasticity, such as task-relevant learning. In this section we present evidence that commonly studied classes of network are, in fact, generically in the vicinity of equilibrium, and that the balance condition (21), far from being an obscure edge case of network configurations, is in some sense a generic state. In particular, we find that the balance condition is approximately attained by any learning algorithm minimizing a very general class of regularized loss functions.

Recall that we denote by $F_{\text{RNN}}\left(W\right)$ the input-output map of the network (3), which takes input trajectories {**u**(*t*)} to output trajectories {**y**(*t*)} and which is parameterized by the weight configuration $W=(W^{\text{in}},J,W^{\text{out}})$. Consider an arbitrary loss function *L* on the input-output map *F*_RNN_. We are not concerned with the particular functional form of *L*—just that it depends on the weight configuration only through the input-output map. For example, the loss might measure the performance of the network on some task, with weight configurations that attain lower loss achieving better task performance.

We consider a regularized loss function *L*_reg_ which is the sum of the loss *L* and element-wise regularization of the recurrent weight matrix:

$$\begin{array}{c} L_{\text{reg}}\left(W\right)=L(F_{\text{RNN}}(W))+\sum_{ij}\alpha_{ij}{\left|J_{ij}\right|}^{p}, \end{array}$$ (26)

where *p*, *α*_*ij*_ > 0 for all *i*, *j*. The expression for *L*_reg_ encompasses both *ℓ*_1_ and *ℓ*_2_ regularization of the recurrent weight matrix, for example, by setting *p* = 1 and *p* = 2 respectively.

**Proposition 4**. *Suppose* *L*_reg_ *has a local minimum at the weight configuration* $W^{*}$. *Then* $W^{*}$ *satisfies the balance condition* (21), *with synaptic costs c*_*ij*_ = *α*_*ij*_|*J*_*ij*_|^*p*^.

*Proof*. We provide a proof by contradiction. Assume that there exists a weight configuration $W^{0}$ which is a local minimum of *L*_reg_ but which is not an equilibrium of synaptic balancing. Let **h**(*t*), *t* ≥ 0, describe the trajectory of synaptic balancing initialized at $W^{0}$, with synaptic costs *c*_*ij*_ = *α*_*ij*_|*J*_*ij*_|^*p*^. Because synaptic balancing preserves the input-output map *F*_RNN_, the loss function $L(F_{\text{RNN}}\left(\pi_{h(t)}\left(W^{0}\right)\right))$ is constant as a function of *t*, i.e.,

$$\begin{array}{c} \frac{d}{dt}L\left(F_{\text{RNN}}\left(\pi_{h(t)}\left(W^{0}\right)\right)\right)=0. \end{array}$$ (27)

Because we have assumed that $W^{0}$ is not an equilibrium of synaptic balancing, the total cost *C* = ∑_*ij*_ *α*_*ij*_|*J*_*ij*_|^*p*^ is strictly decreasing at *t* = 0, i.e.,

$$\begin{array}{c} \frac{d}{dt}C\left(\pi_{h(t)}\left(W^{0}\right)\right)<0. \end{array}$$ (28)

Combining (27) and (28), we have that

$$\begin{array}{c} \begin{array}{cc} \frac{d}{dt}L_{\text{reg}}\left(\pi_{h(t)}\left(W^{0}\right)\right)= & \frac{d}{dt}L\left(F_{\text{RNN}}\left(\pi_{h(t)}\left(W^{0}\right)\right)\right)\\ & +\frac{d}{dt}C\left(\pi_{h(t)}\left(W^{0}\right)\right)\\ < & \;0 \end{array} \end{array}$$

for all *t* and in particular, *t* = 0. Thus, *L*_reg_ is a decreasing function on the trajectory of synaptic balancing initialized at $W^{0}$, and $W^{0}$ is not a local minimum of *L*_reg_. We conclude that every local minimum of *L*_reg_ satisfies the balance condition (21).

An inverse result—that in unregularized networks trained with gradient descent, the initial (generally nonzero) neural gradients are exactly conserved by training—has been noted in, e.g., [42, 43].

**Fig 4:**
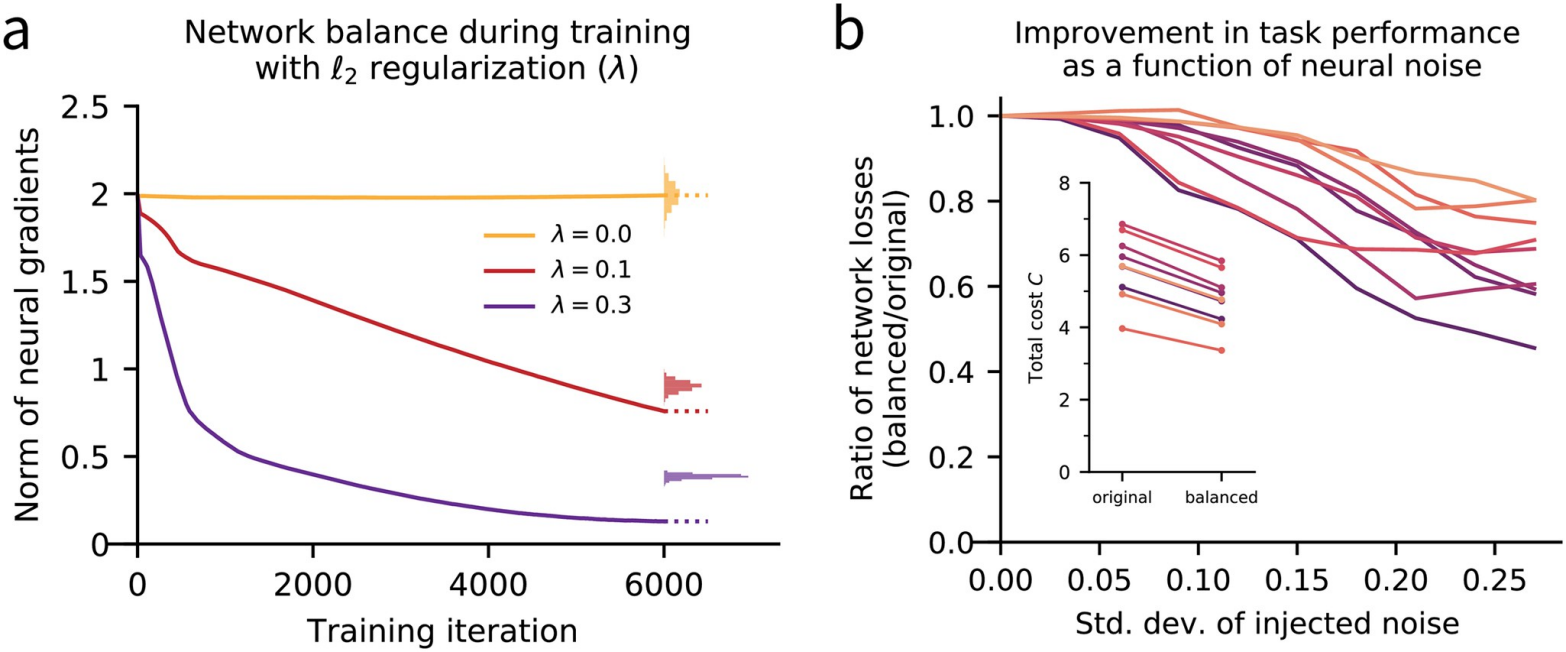
The balance condition in trained networks. **(a)** ReLu networks with *N* = 256 neurons are trained via gradient descent on a context-dependent integration (CDI) task modeled after [23], with varying levels of *ℓ*_2_ penalty $\lambda \sum_{ij}J_{ij}^{2}$. The norm of neural gradients, ‖**g**‖, is shown over the course of training. When λ = 0, neural gradients are fixed by gradient descent dynamics. When λ > 0, trained solutions tend towards the balance condition (21), with $c_{ij}=J_{ij}^{2}$. Histograms at right denote the empirical null distribution of ‖**g**‖ under permutation of the rows of **C** at the final training iteration. For positive values of λ, the actual value of ‖**g**‖ (dotted line) falls significantly below the null distribution. **(b)** Synaptic balancing with the robustness cost function (13) is applied to several ReLu networks trained with λ = 0.3 (corresponding to the purple curve in panel **a**). The original and balanced networks are run on the CDI task at varying levels *ε* of Gaussian noise injected into hidden dynamics. For each network pair the ratio is plotted of task loss of the balanced network to that of the original network, as a function of *ε*. As dynamics become more noisy, the performance of the original networks (as measured by loss on the task) degrades faster than that of the corresponding balanced networks. Inset: total cost *C* of the original vs. balanced networks (12).

In practice, numerical optimization algorithms for network training terminate before achieving true local minima, so we do not expect every network trained with regularization to exactly exhibit the balance condition (21). In Fig 4a we show, however, that trained, *ℓ*_2_-regularized networks exhibit neural gradients that are substantially lower than would be attained by chance, under random perturbation of rows of the weight matrix.

In understanding these results, the reader should keep in mind that under our terminology, a network may be balanced with respect to one cost function (say, the *ℓ*_2_ cost), but not balanced with respect to another cost function (say, the robustness cost). Similarly, the form of regularization that is used during training affects the balance properties of the local minimum reached; a network trained with *ℓ*_2_ regularization need not satisfy the robustness balance condition.

Nonetheless, we have showed that any regularized network will become exactly balanced (in some sense) through training. This observation suggests that attaining the equilibrium condition of synaptic balancing is not only functionally desirable from a robustness standpoint but also is a generic consequence of learning rules which optimize a loss function of the form (26).

### Balancing empirically improves task performance

To experimentally measure the effect of synaptic balancing on task performance, we trained networks via gradient descent on a context-dependent integration task modeled after [23]. This process yielded a trained network which adequately performed the task. We applied synaptic balancing to the trained network, using the robustness cost function (13), and stored the equilibrium weight configuration as our balanced network. We simulated the trajectories of the original (trained) versus balanced network and observed task performance while varying the levels of additive Gaussian noise in the neural dynamics (1). Details of the task and network training are provided in S1 Appendix 5.

As expected, both the original and balanced networks perform identically in the absence of noise, due to the task-preserving nature of synaptic balancing. We also found that the task performance, as measured by the trial-averaged task loss on held-out test data, deteriorated in both the trained and balanced networks as the noise level increased. However, this deterioration was noticeably attenuated in the balanced networks, and trained networks that had not been balanced proved more sensitive to higher levels of Gaussian noise. The decay in relative performance of original versus balanced networks is illustrated across several network instantiations in Fig 4b.

The improvement exhibited by the balanced network is remarkable in part because synaptic balancing does not make use of a task-specific error signal, but merely uses summary statistics of the average neuronal gain during the task. These results confirm that in networks which are already performing a task, the variability of neural responses can be suppressed simply by shifting synaptic weights towards a balanced configuration via the task-preserving transformation. As noted above, this task preserving transformation can be implemented by local synaptic learning rules that require no knowledge of the task.

### Exact and approximate trajectories of synaptic balancing

We have shown that the ordinary differential Eq (18) is a member of a widely studied class of dynamical systems called Lax dynamics. In the interest of better understanding the action of our dynamics on the recurrent weight matrix as a whole, we now turn to closed-form solutions to (18) that specify the evolution of synaptic balancing over time. When the network has just two neurons, our solution is exact; in general, we derive a quadratic approximation taking the form of a heat equation.

#### Exact trajectory with two neurons

In a two-neuron network, the balancing dynamics admit an exact analytical solution for the time course of the synaptic costs *c*_12_ and *c*_21_, when these costs take the power-law form (23). If both initial synaptic costs are positive, we find that they evolve as

$$\begin{array}{c} \begin{array}{c} c_{12}\left(t\right)={\hat{c}}_{12}\;q\left(t\right)\\ c_{21}\left(t\right)={\hat{c}}_{12}\;q{\left(t\right)}^{-1}, \end{array} \end{array}$$ (29)

where

$$\begin{array}{c} {\hat{c}}_{ij}=\sqrt{c_{ij}^{0}c_{ji}^{0}} \end{array}$$ (30)

and

$$\begin{array}{c} q\left(t\right)=\text{tanh}\;[2p^{2}\left({\hat{c}}_{12}\right)t+{\text{tanh}}^{-1}{\left(c_{12}^{0}/c_{21}^{0}\right)}^{1/2}] \end{array}$$

If just a single initial synaptic cost is positive—suppose it is *c*_12_—then it evolves as

$$\begin{array}{c} c_{12}\left(t\right)=\frac{c_{12}^{0}}{2c_{12}^{0}\gamma p^{2}\;t+1} \end{array}$$ (31)

while *c*_21_ is fixed at zero. In agreement with Prop. 3, the latter case suffers from unbounded growth of the input and output weights over time as *J*_12_ → 0, and there is no stable equilibrium of the dynamics. The trajectories in (29) and (31) are illustrated in panels (g-h) of Fig 3.

#### Approximate trajectory via the heat equation

Except in the two-neuron scenario, we are not aware of a general analytic solution to the trajectory of synaptic balancing. Here we show that a closed-form approximate solution is attainable in general, however, shedding light on the macroscopic patterns of weight modification that synaptic balancing induces in a network. Our approach is to consider the time evolution of the neural gradients, which we tie to solutions of the heat equation on a graph. This then yields a closed-form approximate local solution for the trajectory of the coordinates **h**.

The time evolution of the neural gradients (17) is given by

$$\begin{array}{c} \dot{g}=\frac{\partial g}{\partial h}g. \end{array}$$ (32)

Since **h** evolves under gradient descent (15), the Jacobian of the dynamics of **h** is a sign-flipped version of the Hessian of the cost function (12),

$$\begin{array}{c} \frac{\partial g}{\partial h}=-\gamma \frac{\partial^{2}C}{\partial h^{2}}. \end{array}$$ (33)

A short calculation via (16) finds the Hessian to be

$$\begin{array}{c} \frac{\partial^{2}C}{\partial h^{2}}=p^{2}L, \end{array}$$ (34)

where **L** takes the form of a Laplacian matrix corresponding to a graph with edge weights given by what we call the conductance matrix $\bar{C}$, which has *ij*th element

$$\begin{array}{c} {\bar{c}}_{ij}=c_{ij}+c_{ji}, \end{array}$$ (35)

and which is evidently symmetric. Explicitly, the Laplacian **L** has elements drawn from $\bar{C}$ as follows:

$$\begin{array}{cc} L_{ij} & =\{\begin{array}{cc} -{\bar{c}}_{ij}, & i\neq j,\\ \sum_{k\neq i}{\bar{c}}_{ki}, & i=j. \end{array} \end{array}$$ (36)

Combining (32), (33) and (34), and maintaining *γ* = *p*^−1^ as before, the time derivative of the neural gradients is

$$\begin{array}{c} \dot{g}=-pLg. \end{array}$$ (37)

Eq (37) is the heat equation of the graph corresponding to **L**, in analogy to the heat equation in continuous environments [44]. This suggests an intuitive physical description, and approximate mathematical solution, of the evolution of neural gradients.

Since **L** is itself time-varying, the dynamics (37) do not admit a straightforward exact solution. However, in the vicinity of a weight configuration $\tilde{W}$, we may take **L** to be fixed at $\tilde{L}$ to obtain a closed-form expression which approximates the exact solution to (37). This corresponds to gradient descent dynamics on the quadratic Taylor approximation of *C* in **h** evaluated at $\tilde{W}$. Under fixed $\tilde{L}$, (37) is a (time-invariant) linear dynamical system, and the solution at time *t* may be expressed in terms of the heat kernel $e^{-p\tilde{L}t}$:

$$\begin{array}{cc} g(t) & =e^{-p\tilde{L}t}g^{0}\\ & =\sum_{i:\lambda_{i}>0}e^{-p\lambda_{i}t}v^{i}{\left(v^{i}\right)}^{T}g^{0}, \end{array}$$ (38)

where **g**^0^ denotes the neural gradient at time *t* = 0, and λ_*i*_, **v**^*i*^ denote the *i*th eigenvalue and eigenvector of $\tilde{L}$. The sum is taken over all *i* such that λ_*i*_ is positive, since if λ_*i*_ = 0 for some *i* then **v**^*i*^ is an indicator vector of a connected component, which must be orthogonal to any realizable neural gradient **g**^0^.

Integrating (38) yields a closed-form approximate expression for the evolution of the coordinates **h** on the task-preserving manifold:

$$\begin{array}{c} h(t)=\sum_{i:\lambda_{i}>0}{\left(p\lambda_{i}\right)}^{-1}\left(1-e^{-p\lambda_{i}t}\right)v^{i}{\left(v^{i}\right)}^{T}g^{0}, \end{array}$$ (39)

where we have chosen boundary conditions such that **h**(0) = 0. As *t* → ∞, the network reaches an equilibrium **h** → h*, given by

$$\begin{array}{c} h^{*}=p^{-1}{\tilde{L}}^{†}g^{0}. \end{array}$$ (40)

The notation ${\tilde{L}}^{†}$ denotes the Moore-Penrose pseudoinverse of $\tilde{L}$.

**Fig 5:**
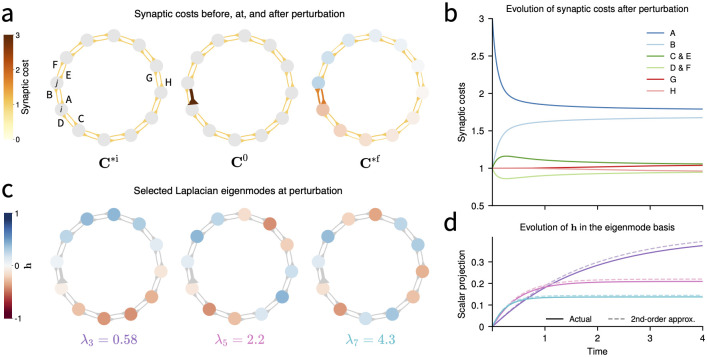
Dynamics of synaptic balancing in a 12-neuron ring network with single perturbed synapse. **(a)**. Left: A ring network is at an initial equilibrium C*^i^ with all synaptic costs equal to 1. Nodes indicate neurons; arrows indicate directed synapses. Center: Synapse A, from neuron *j* to *i*, is instantaneously potentiated to a synaptic cost of $c_{ij}^{0}=3$. Right: Synaptic balancing relaxes to a new equilibrium C*^f^. Synapse colors and thickness indicate synaptic cost. Neuron colors indicate value of h*^f^ according to color scheme of panel (c). **(b)** Time course of synaptic balancing following perturbation. Incoming synapses to neuron *i* (A and D) are weakened and outgoing synapses from neuron *i* (B and C) are strengthened. Incoming synapses to neuron *j* (B and E) are strengthened and outgoing synapses from neuron *j* (A and F) are weakened. Synapses (H and G) that are distant from the site of perturbation respond more slowly than proximate synapses though they reach the same equilibrium values. **(c)** Three eigenmodes **v**_3_, **v**_5_, **v**_7_, with eigenvalues λ_3_, λ_5_, λ_7_, of the Laplacian matrix **L**^0^ corresponding to the conductance matrix at the moment of perturbation. Color indicates mode value at each neuron. **(d)** Dynamics of **h** approximately decompose into the basis of Laplacian eigenmodes. The scalar projection of **h** onto each mode **v** is shown along with the quadratic approximation (39), using **L**^0^ as Laplacian. Line color matches eigenvalue color in panel (c).

To illustrate these concepts, Fig 5 shows how a 12-neuron, ring topology network, redistributes synaptic cost by synaptic balancing dynamics in response to a perturbation of a single synaptic cost (a—b), as well as the evolution of **h** in the Laplacian matrix eigenmode basis (c-d). The approximate closed-form dynamics derived in this section, and especially the role of the Laplacian matrix **L**, help shape intuition about the behavior of our rule: Neural gradients diffuse in a network akin to heat diffusing on a graph, with higher spatial-frequency modes of the graph decaying more quickly than lower spatial-frequency modes. As shown in Fig 5(d), numerical simulations verify that the quadratic approximate dynamics presented here accurately describe synaptic trajectories near equilibrium.

#### General bounds on equilibrium cost

We have provided exact and approximate closed-form dynamics of synaptic balancing. To complement these results, we now turn to results on the optimal value of the cost function, stated in terms of the initial cost matrix **C**^0^.

**Proposition 5**. *Suppose synaptic costs are of the power-law form* (23). *Then given initial neural gradients* **g**^0^ *and initial total cost C*^0^, the minimum total cost C* *on the task-preserving manifold is bounded above by*

$$\begin{array}{c} C^{*}\leq C^{0}-\frac{1}{8C^{0}}{\left\|g^{0}\right\|}_{2}^{2}, \end{array}$$ (41)

*and below by*

$$\begin{array}{c} \sum_{ij}{\hat{c}}_{ij}\leq C^{*}, \end{array}$$ (42)

*where* ${\hat{c}}_{ij}=\sqrt{c_{ij}^{0}c_{ji}^{0}}$ *for all i*, *j*, *as in* (30).

A proof is given in S1 Appendix. Intuitively, the upper bound (41) says that the greater the initial deviation from the balance condition (in the form of large neural gradients), the greater the guarantee that synaptic balancing will improve the total cost. The lower bound (42) says that the more symmetric the initial cost matrix, the less that synaptic balancing is able to improve the costs.

Recall that in a two-neuron network, the equilibrium cost matrix is symmetric (29). In fact, we find that symmetry is preferred by synaptic balancing, and the equilibrium cost matrix will be symmetric in any circumstance in which symmetry is attainable on the task-preserving manifold. If **C**^0^ is the initial matrix of synaptic costs, and **C**^0^ is positive diagonally symmetrizable, then the minimum is attained at the cost matrix $\hat{C}=e^{-H^{*}}C^{0}e^{H^{*}}$, whose *i*, *j*th element is the geometric mean of initial costs (30), and equality is attained in (42).

As an example of symmetric equilibrium beyond the case of *N* = 2, consider a rank-one network whose initial costs factor as **C**^0^ = **ab**^*T*^. This network is strongly connected only if every element of **a** and **b** is positive, in which case the equilibrium cost matrix is C* = a*b*^T^, where $a_{i}^{*}=b_{i}^{*}=\sqrt{a_{i}b_{i}}$.

It is not just symmetric matrices which are fixed points of synaptic balancing: all normal matrices—including symmetric matrices as a special case—are equilibria of our rule, i.e., they satisfy the balance condition (21). This more general result is shown in S1 Appendix.

In purely linear networks, arbitrary similarity transformations of the recurrent matrix—not just positive diagonal transformations—are task-preserving. The positive diagonal similarity transformation (4), then, will generically not achieve the optimal sensitivity among task-equivalent *linear* networks. It would be interesting to explore how optimizing over similarity transforms involving all invertible matrices might perform relative to our purely diagonal similarity transformation. However, we note that such an optimization will not generically lead to a biologically plausible local learning rule.

### Synaptic balancing predicts heterosynaptic plasticity

Previous sections demonstrated that the balance condition (21) is not only functionally useful for noise robustness but also in a sense generic in trained, regularized recurrent networks. These findings suggest that synaptic balance may be a widespread phenomenon, with many networks attained through regularized training naturally exhibiting the equilibrium condition of synaptic balancing (21). In a biological context, where we hypothesize that synaptic balancing would continually operate alongside other weight dynamics, we predict that the network is generically in a state fluctuating around the balance condition. For example, fast-acting, inherently unstable plasticity rules—Hebbian or otherwise—might temporarily bring synapses and firing rates away from equilibrium, while synaptic balancing slowly tunes the network in response to those modifications, so that the balance condition is always approximately maintained.

This suggests that synaptic balancing might most commonly play a role in fine-tuning networks that are already in the vicinity of a balanced equilibrium. To explore this scenario in a specific, experimentally testable regime, we consider the response of synaptic balancing to a single synaptic potentiation or depression near equilibrium, as depicted for a ring network case in Fig 5.

#### Synaptic perturbations induce compensatory heterosynaptic plasticity

Suppose the that network begins at an initial equilibrium configuration $W^{*i}$ satisfying the balance condition (21), and that a perturbation is applied. Specifically, let the *i*, *j*th synapse (*i* ≠ *j*) be instantaneously modified by a small factor *η* ≈ 0 while every other synapse is unchanged. Call the perturbed weight configuration $W^{0}$. Then

$$\begin{array}{c} J_{kl}^{0}=J_{kl}^{*\text{i}}\left(1+\eta \delta_{ik}\delta_{jl}\right). \end{array}$$ (43)

If *η* > 0, this perturbation corresponds to synaptic potentiation of *J*_*ij*_, and if *η* < 0, it corresponds to synaptic depression.

By first-order expansion of the power-law synaptic costs (23), we have that the costs adjust as

$$\begin{array}{c} c_{kl}^{0}=c_{kl}^{*\text{i}}\left(1+\eta p\delta_{ik}\delta_{jl}\right). \end{array}$$ (44)

Plugging the perturbed synaptic costs (44) into the expression for the neural gradient (17), and using that the network was initialized at equilibrium, i.e. that $g_{k}^{*\text{i}}=0$ for all *k*, we have,

$$\begin{array}{c} \begin{array}{c} g_{k}^{0}=\eta pc_{ij}^{*\text{i}}\left(\delta_{ik}-\delta_{jk}\right). \end{array} \end{array}$$ (45)

Finally, by plugging (43) and (45) into (18), the learning rule becomes, to first order,

$$\begin{array}{c} {\dot{J}}_{kl}=\eta pJ_{kl}^{*i}c_{ij}^{*i}\left(\delta_{il}-\delta_{jl}+\delta_{jk}-\delta_{ik}\right) \end{array}$$ (46)

This rule predicts that a perturbation from equilibrium at a single synapse from *j* to *i* will lead to multiplicative plasticity at the outgoing and incoming synapses of both neurons. Specifically, if *J*_*ij*_ is potentiated, i.e., if *η* > 0, then the response of synaptic balancing is potentiation of the presynaptic neuron’s incoming synapses (*J*_*jl*_) and of the postsynaptic neurons’ outgoing synapses (*J*_*ki*_), as well as depression of the presynaptic neuron’s outgoing synapses (*J*_*kj*_) and the postsynaptic neuron’s incoming synapses (*J*_*il*_).

Following the perturbation from the initial equilibrium configuration $W^{*i}$, the system will relax under synaptic balancing to a final equilibrium configuration $W^{*f}$ (Fig 5(a) and 5(b)). To calculate the change in synaptic strength of neuron *ij* from its initial equilibrium to its final equilibrium value, we plug the value of **g**^0^ from (45) into (40) to find

$$\begin{array}{c} h^{*f}=\eta c_{ij}^{*i}L^{†}\left(e_{i}-e_{j}\right), \end{array}$$ (47)

where **e**_*k*_ is the *k*th standard basis vector of $R^{N}$, and we adopt the Laplacian matrix corresponding to the perturbed weight configuration $W^{0}$. Writing the task-preserving transformation $J_{ij}^{*f}=J_{ij}^{0}e^{h_{j}^{*f}-h_{i}^{*f}}$ in terms of (47), then the log change in synaptic weight, normalized by the perturbation *η*, is

$$\begin{array}{c} \begin{array}{cc} \frac{1}{\eta }\text{log}(\frac{J_{ij}^{*f}}{J_{ij}^{0}}) & =-c_{ij}^{0}\left({\left(L^{†}\right)}_{ii}+{\left(L^{†}\right)}_{jj}-2{\left(L^{†}\right)}_{ij}\right)\\ & =-c_{ij}^{0}R_{ij}^{0} \end{array} \end{array}$$ (48)

where $R_{ij}^{0}={\left(L^{†}\right)}_{ii}+{\left(L^{†}\right)}_{jj}-2{\left(L^{†}\right)}_{ij}\geq 0$ is the resistance distance (alternately called effective resistance) between neurons *i* and *j*, on the graph whose edges have conductances ${\bar{C}}^{0}$. Resistance distance generalizes to arbitrary graphs the formulas for resistance in series and parallel, and *R*_*ij*_ is greater where the paths of conductivity between neurons *i* and *j* are fewer or weaker [45]. We may interpret (48) to mean that synaptic balancing counteracts a potentiation or depression of *J*_*ij*_ by a factor equal to the fraction of overall conductance between neurons *j* and *i* that is attributable to the synaptic cost $c_{ij}^{0}$, versus to other paths of synaptic costs in the network.

#### Slow, compensatory, and network-wide heterosynaptic plasticity

We have described synaptic balancing near equilibrium as a slow, compensatory mechanism that adjusts a neuron’s input and output synapses in response to a single potentiation or depression, in a manner closely resembling known phenomena of heterosynaptic plasticity [12, 13]. Here we discuss experimental evidence in connection with the predictions of synaptic balancing.

Studies inducing Hebbian plasticity at one or more target synapses have repeatedly observed compensatory heterosynaptic modification at nearby synapses on the same dendrite [14–16, 46, 47]. For example, two-photon *in vivo* imaging of synaptic spines revealed that heterosynaptic depression of inputs to mouse V1 layer 2/3 pyramidal neurons follows functionally induced potentiation (LTP) of synapses on the same dendrite—a result exactly predicted by Eq (46).

Our specific predictions about the magnitude and direction of heterosynaptic effects also find experimental support. Eq (46) predicts heterosynaptic modifications that are multiplicative, with the change in weight proportional to the original size of the synapse, and independent of the synapse’s sign (i.e., inhibitory or excitatory). Patch-clamp recordings of synapses in cortical slice after induction of spike-timing-dependent Hebbian plasticity in paired synapses matched the multiplicative principle, with the heterosynaptic effect applying more strongly to the initially larger unpaired synapses than to the initially smaller ones [16]. The same work found that heterosynaptic effects could be explained solely in terms of the absolute strengths of the paired and unpaired synapses: both E and I synapses experienced compensatory heterosynaptic depression when the paired synapse was potentiated, and both E and I unpaired synapses experienced compensatory potentiation when the paired synapse was depressed.

Our rule may be distinguished from existing concepts of compensatory heterosynapstic plasticity [13, 28] through a further prediction, to our knowledge not yet experimentally explored: in response to the potentiation of a single neuron’s inputs, not only do input synapses experience heterosynaptic depression, but also output synapses experience potentiation. This concept is demonstrated in Fig 5 for a 12-neuron ring network that experiences an instantaneous synaptic perturbation.

Mechanisms supporting the input-synapse half of our conjectured balancing dynamics are well studied. In addition to the known heterosynaptic effects already mentioned, neurons are known to multiplicatively and bidirectionally scale all incoming synapses through synaptic scaling [25, 26, 48, 49]. While the particular homeostatic trigger posited by synaptic scaling—deviations from a set-point firing rate—differs from the trigger considered in our model, synaptic scaling lends evidence to the hypothesis that a neural mechanism exists to distribute a negative feedback signal to incoming synapses and induce coordinated compensatory plasticity, such as is predicted by synaptic balancing.

This work introduces a new view on the possible functional roles of compensatory heterosynaptic plasticity. Existing literature has largely focused on the important role it may play in constraining the inherent instability of Hebbian plasticity [12, 13, 28, 50]. In our model, the role of heterosynaptic plasticity is to maintain a functional state of noise robustness even as other learning processes homosynaptically modify synapse strength. We believe that these traits are suitably thought of as a novel model of homeostatic plasticity: one whose aim is to maintain, through negative feedback, the functionally-relevant balance condition (21).

## Methods

### Network training details

We constructed a context-dependent integration task with three input variables: the context **a** and two signals **s**^(1)^ and **s**^(2)^. Each variable was encoded as a pair of indicator-style inputs, yielding six dimensions of network inputs. Each pair (*a*_1_, *a*_2_), $\left(s_{1}^{\left(1\right)},s_{2}^{\left(1\right)}\right)$, and $\left(s_{1}^{\left(2\right)},s_{2}^{\left(2\right)}\right)$ independently took the value (0, 1) or (1, 0), plus Gaussian noise, for the duration (*T* = 50) of each trial. Three Boolean variables corresponded to eight total trial conditions.

The task target **z** (also encoded as indicator variables *z*_1_, *z*_2_) was the integral of the noisy signal over time, as gated by the context:

$$\begin{array}{c} z_{i}\left(t\right)=\{\begin{array}{cc} \int_{0}^{t}s_{i}^{\left(1\right)}\left(t^{\prime }\right)dt^{\prime }, & a=(0,1),\\ \int_{0}^{t}s_{i}^{\left(2\right)}\left(t^{\prime }\right)dt^{\prime }, & a=(1,0). \end{array} \end{array}$$

for *i* = 1, 2.

Networks of the form (1), with rectified linear activation functions, were initialized with i.i.d. weights $J_{ij}∼N\left(0,N^{-1}\right)$ and were trained via gradient descent to minimize an objective consisting of the squared-error loss function ∑_*i*,*t*_(*y*_*i*_(*t*) − *z*_*i*_(*t*))^2^, plus a regularization term $\lambda \sum_{ij}\;J_{ij}^{2}$. Networks in Fig 4, with *N* = 256 neurons, learned the task after being trained with a fixed learning rate of 0.003 for 1,600 training iterations.

Average gains ${\left\{\sigma_{j}^{2}\right\}}_{j=1}^{N}$ were calculated across conditions and trials on a separate trial dataset not used in the training or evaluation of the network. The balanced network was taken to be the final time step of a numerical solution to the ODE (18), using neural gradients (17) and synaptic costs (13), with the original (trained) network as initial condition. Fig 4b was attained by running the original and balanced networks with varying levels of additive Gaussian noise injected into the recurrent neural dynamics.

## Discussion

Lastly, we would like to discuss some important observations, consequences, and limitations of this work. One particularly notable drawback of this work is the lack of any theoretical guarantees of improvement in the robustness to noise of the output trajectory {**y**(*t*)} to fluctuations in either the input trajectory or the internal units. Instead, we study the sensitivity of the neural dynamics to noise in the internal units, a quantity that is generally considered crucial to task performance but allows us to study noise robustness in a task-agnostic way. This paper has intentionally focused on the case of recurrent networks, where task performance is dominated by the compounded dynamics of the recurrent matrix feeding into itself over time. In the limit of large trial time, we postulate that it is these recurrent dynamics which contribute most essentially to task performance. In this light, we believe the apparent feedforward structure of **W**^in^ → **J** → **W**^out^ distracts from the essential computations at hand. In other words, since noise in the recurrent dynamics leads to compounding errors over time, we expect that the sensitivity of the neural dynamics *S*^**x**→**x**^ (using the notation of section) matters more than *S*^**x**→**y**^ in the limit of large trial time.

As we show that regularized networks lead to more balanced solutions, one may ask if a biological system would be better off implementing a form of regularization rather than our local synaptic update rule. Synaptic balancing explores a limited manifold with *N* degrees of freedom, where the underlying input-output function is fixed. In contrast, network training may optimize over the entire input, recurrent, and output matrices. Even highly regularized loss functions, which effectively constrain the degrees of freedom available to the optimization, allow the network to optimize over different input-output functions. Our analysis of synaptic balancing as a dynamical system reveals a connection between learning in noise-robust systems and heterosynaptic plasticity. This connection is new to our knowledge and merits further investigation. Furthermore, we believe our learning rule will be significantly better than weight decay after a task is learned and no more training data for that task is arriving to further maintain task performance (i.e. our memory of how to ride a bike when we haven’t ridden a bike in some time). If we use weight decay in the post learning regime, then weight decay will clearly destroy task performance (we will forget how to ride bikes during periods when we are not riding bikes). In contrast, a task-preserving plasticity rule, such as synaptic balancing, would move trained circuits to solutions that are more robust to internal noise even when the task is not actively being trained without destroying any learned information (causing us to forget how to ride a bike).

Lastly, the crucial aspects of synaptic balancing that it provably preserves the exact input-output relation and to do so explores a limited manifold with *N* degrees of freedom has consequences for the efficacy of this synaptic update rule for high-dimensional networks. We show in appendix Fig 1 that the relative reduction in total cost (a proxy for sensitivity on the task-preserving manifold) decreases with the rank of the initial synaptic weight matrix. It is possible that this effect of modest improvements for high-dimensional networks could be mitigated by relaxing this requirement slightly, and transforming the network in a way that only approximately preserves the task. Perhaps an approximate rule inspired by this work could result in larger improvements in robustness as a function of initial rank, while any damage done to the task performance could be corrected with a small amount of retraining. We believe this would be an interesting future research direction.

## Summary

In this work, we introduced a positive diagonal similarity transformation of the recurrent weight matrix that preserves task performance in nonlinear recurrent networks with homogeneous nonlinearities. We showed that a simple class of cost functions, notably including the sensitivity of the neural dynamics to noise, are convex in the coordinates of the symmetry. From this observation, we derived a local learning rule—synaptic balancing—that globally maximizes network robustness whenever the recurrent network is strongly connected.

We found that the synaptic cost matrix is balanced at equilibrium, and that this balance condition arises at every local minimum of a very general class of regularized loss functions. To further understand how synaptic balancing dynamics shape network connectivity in the vicinity of equilibrium, we approximated the dynamics of our rule through a heat equation and described the diffusion of synaptic modifications throughout the network according to its Laplacian eigenmodes. We found that near equilibrium, synaptic balancing is well summarized as slow, compensatory, and heterosynaptic, and that experimental evidence of heterosynaptic plasticity is consistent with our predictions.

Overparameterization in neural network models may be linked to the biological processes which sustain task performance under noisy conditions—a possibility known to experimental neuroscience for some time [51]. Here, we have provided a concrete, analytically tractable example of this concept, in which an identifiable symmetry in network parameterization gives rise to a corresponding local process for maintaining stable task performance. We hope that this work may provide a fruitful framework for future research relating homeostatic processes to the mathematical structures underpinning neural network redundancy.

## Supporting information

S1 Appendix Additional proofs. Full proofs of Propositions 1, 3 and 5.
(PDF)
