# Supplementary material for: Synaptic balancing: A biologically plausible local learning rule that provably increases neural network noise robustness without sacrificing task performance
Source: PLoS Comput Biol. 2022 Sep 19;18(9):e1010418. doi: 10.1371/journal.pcbi.1010418 (PMC9522011; doi:10.1371/journal.pcbi.1010418)
Supplement: S1 Appendix — Full proofs of Propositions 1, 3 and 5. (PDF) [file pcbi.1010418.s001.pdf]

S1 Appendix

Accompanies *Synaptic balancing: a biologically plausible local learning rule that provably increases neural network noise robustness without sacrificing task performance*, by Christopher H. Stock, Sarah E. Harvey, Samuel A. Ocko, and Surya Ganguli.

1 The TPT is task-preserving

Proposition 1. *[Task-preserving transformation] The transformation* (4) *exactly preserves the input-output relationship of the neural dynamics* (1)*. Given two networks receiving the same time course of inputs* $\mathbf{u}(t)$ *and with weight configurations* $\mathcal{W}^0$ *and* $\mathcal{W} = \pi_{\mathbf{h}}(\mathcal{W}^0)$ *respectively, then:*

1. *If* $\mathbf{x}^0(t)$ *is the time course of hidden unit neural activity under* $\mathcal{W}^0$*, then* $e^{-\mathbf{H}}\mathbf{x}^0(t)$ *is the time course of hidden unit neural activity under* $\mathcal{W}$*.*
2. *If* $\mathbf{y}^0(t)$ *is the time course of output neural activity under* $\mathcal{W}^0$*, then* $\mathbf{y}^0(t)$ *is also the time course of output neural activity under* $\mathcal{W}$*.*

Proof. From (1), the dynamics of the neural activity $\mathbf{x}$ in the network with transformed weights $\mathcal{W}$ is

$$\mathbf{f}_{\mathcal{W}}(\mathbf{x}, \mathbf{u}) = -\mathbf{x} + e^{-\mathbf{H}}\mathbf{J}^0 e^{\mathbf{H}}\phi(\mathbf{x}) + e^{-\mathbf{H}}\mathbf{W}^{\text{in},0}\mathbf{u}.$$

Multiplying on the left by $e^{\mathbf{H}}$, and using that ϕ is homogeneous, we have

$$\begin{aligned} e^{\mathbf{H}}\mathbf{f}_{\mathcal{W}}(\mathbf{x}, \mathbf{u}) &= -e^{\mathbf{H}}\mathbf{x} + \mathbf{J}^0\phi(e^{\mathbf{H}}\mathbf{x}) + \mathbf{W}^{\text{in},0}\mathbf{u} \\ &= \mathbf{f}_{\mathcal{W}^0}(\mathbf{z}, \mathbf{u}), \end{aligned} \tag{S.1}$$

where $\mathbf{z} = e^{\mathbf{H}}\mathbf{x}$. The time integral of the left hand side is the scaled neural activity of the network with weights $\mathcal{W}$:

$$\begin{aligned} \int_0^t e^{\mathbf{H}}\mathbf{f}_{\mathcal{W}}(\mathbf{x}, \mathbf{u})\, \mathrm{d}t' &= e^{\mathbf{H}} \int_0^t \mathbf{f}_{\mathcal{W}}(\mathbf{x}, \mathbf{u})\, \mathrm{d}t' \\ &= \tau e^{\mathbf{H}}\mathbf{x}(t), \end{aligned} \tag{S.2}$$

using that $\mathbf{x}(0) = 0$. The time integral of the right hand side of (S.1) is the neural activity of the network with weights $\mathcal{W}^0$:

$$\int_0^t \mathbf{f}_{\mathcal{W}^0}(\mathbf{z}, \mathbf{u})\, \mathrm{d}t' = \tau\mathbf{x}^0(t). \tag{S.3}$$

Equating (S.2) and (S.3), we find that

$$\mathbf{x}(t) = e^{-\mathbf{H}}\mathbf{x}^0(t), \tag{S.4}$$

demonstrating the first part of the proposition.

From (2) and (S.4), we have that the readout of the transformed network is

$$\begin{aligned}
\mathbf{y}(t) &= \mathbf{W}^{\text{out}}\mathbf{x}(t) \\
&= (\mathbf{W}^{\text{out},0}e^{\mathbf{H}})(e^{-\mathbf{H}}\mathbf{x}^0(t)) \\
&= \mathbf{W}^{\text{out},0}\mathbf{x}^0(t) \\
&= \mathbf{y}^0(t),
\end{aligned}$$

demonstrating the second part of the proposition. □

2 Existence and stability of equilibria

In this section we prove that under reasonable assumptions on the topology of the network, a minimum-cost weight configuration exists on the task-preserving manifold and is exponentially stable under synaptic balancing. The results proved in this section imply Prop. 3 of the main text.

We begin with some definitions related to network topology. A connected component of the network refers to a connected component of the undirected graph whose edge weights are given by the (symmetric) conductance matrix $\bar{\mathbf{C}}$ (35). Further, a set of neurons $\mathcal{K}$ is strongly connected if and only if for every $i, j \in \mathcal{K}$ one may follow a path of positive (directed) synaptic costs from i to j. Under the task-preserving transformation, synaptic costs which are initially zero remain zero, and costs which are initially positive remain positive, so topological properties of the network wiring diagram do not vary over the course of synaptic balancing. Throughout the appendix we assume synaptic costs are of the power-law form (23), unless explicitly stated otherwise.

2.1 Total cost attains a global minimum on the task-preserving manifold in strongly connected networks

We now establish a lemma on the boundedness of sublevel sets of the total cost C in strongly connected networks.

As a preliminary comment, we generalize the observation made in the main text that $\sum_i h_i(t) = 0$ for all $t \geq 0$ (22). In particular, if $\mathcal{K}$ is a connected component of the network, then $\sum_{k\in\mathcal{K}} g_k = 0$ for every synaptic cost matrix. This may be seen by writing out g_k in terms of synaptic costs (17) and using that $c_{ij} = 0$ if neurons i and j are in different connected components. If a network has K connected components and $\mathbf{k}_1, \mathbf{k}_2, \ldots, \mathbf{k}_K \in \mathbb{R}^N$ are indicator vectors for each component, then for each $i = 1, 2, \ldots, k$,

$$\mathbf{k}_i^T\mathbf{h} = \mathbf{k}_i^T\mathbf{h}^0 = 0, \tag{S.5}$$

since $\mathbf{g} = \dot{\mathbf{h}}$.

Lemma 1. *Suppose that every connected component of a network is strongly connected, and that the network evolves under synaptic balancing from an initial total cost C^0. Then there exists some compact box $\mathcal{B} \subset \mathbb{R}^N$ which contains the sublevel set $\{\mathbf{h} : C(\mathbf{h}) \leq C^0\}$.*

Proof. We give an intuitive upper bound on the individual synaptic costs, and we telescope it along paths of synapses in a strongly connected network to show that the sublevel set of C^0 is bounded.

To approximate the set of coordinates $\mathbf{h}$ such that $C \leq C^0$, we first note that a simple upper bound on the synaptic cost c_{ij} for weight configurations in the sublevel set $\{\mathbf{h} : C(\mathbf{h}) \leq C^0\}$ is

$$c_{ij} \leq C^0, \tag{S.6}$$

since $c_{ij} \leq C$ and by assumption $C \leq C^0$. Using that $c_{ij} = c^0_{ij} e^{p(h_j - h_i)}$ (14), we solve for $e^{h_j - h_i}$ in (S.6), assuming $c^0_{ij} \neq 0$:

$$e^{h_j - h_i} \leq (C^0 / c^0_{ij})^{1/p}. \tag{S.7}$$

To obtain an analogous upper bound when $c_{ij} = 0$, now let m and n any be two neurons in the same connected component of the network. By the strongly connected assumption, there is some path of neurons $(m, i_1, i_2, \ldots, i_\kappa, n)$ from m to n such that each consecutive directed synapse along the path has positive synaptic cost. Telescoping (S.7) along this path, we obtain

$$\begin{aligned} e^{h_m - h_n} &= e^{h_m - h_{i_1}} e^{h_{i_1} - h_{i_2}} \cdots e^{h_{i_\kappa} - h_n} \\ &\leq (C^0 / c^0_{i_1 m})^{1/p} (C^0 / c^0_{i_2 i_1})^{1/p} \cdots (C^0 / c^0_{n i_\kappa})^{1/p}. \end{aligned}$$

Then apply this procedure along a path from neuron n to neuron m to obtain an upper bound for $e^{h_n - h_m}$.

The upper bounds for $e^{h_m - h_n}$ and $e^{h_n - h_m}$ imply that $|h_m - h_n|$ is bounded above for every m, n in a shared a connected component. Combined with the constraint (S.5), it follows that $|h_m|$ is bounded above for all neurons m, and the sublevel set $\{\mathbf{h} : C(\mathbf{h}) \leq C^0\}$ is contained within some box $\mathcal{B} \subset \mathbb{R}^N$. □

The previous lemma may be strengthened into a general result giving sufficient and necessary conditions on the existence of equilibria of synaptic balancing. The following result is known [1], [2]; for completeness we state and prove it in the language of this paper.

Proposition 1. *The total cost C (12) attains a global minimum on the task-preserving manifold if and only if every connected component of the network is strongly connected.*

Proof. In the first direction, assume that there is at least one pair of neurons m, n in a connected component $\mathcal{K}$ such that n cannot reach m through a directed path of positive synaptic costs. Our approach is to show that there is no weight configuration with this topology that can satisfy (21).

Suppose that a connected component of the network is partitioned into two sets of neurons $\mathcal{I}$ and $\mathcal{J}$. We sum (21) over $k \in \mathcal{I}$ and use the fact that $c_{kl} = c_{lk} = 0$ if neurons k and l are in different connected components to obtain

$$\sum_{i\in\mathcal{I},j\in\mathcal{J}} c_{ji} = \sum_{i\in\mathcal{I},j\in\mathcal{J}} c_{ij}. \tag{S.8}$$

In short, if C is minimized, then aggregate costs from $\mathcal{I}$ to $\mathcal{J}$ are equal to the aggregate costs from $\mathcal{J}$ to $\mathcal{I}$.

Let $\mathcal{I} \subset \mathcal{K}$ be the set of neurons which can reach m through a directed path of synaptic costs, and let $\mathcal{J} \subset \mathcal{K}$ be set of neurons which cannot. The sets $\mathcal{I}$ and $\mathcal{J}$ are not empty; they include neurons m and n respectively. On one hand, the synaptic cost c_{ij} must be zero for every $i \in \mathcal{I}$ and $j \in \mathcal{J}$ (else j could reach m). On the other hand, at least one cost c_{ji}, for some $i \in \mathcal{I}$ and $j \in \mathcal{J}$, must be greater than zero (else $\mathcal{I}$ and $\mathcal{J}$ would be in different connected components). It is impossible for the total synaptic costs from $\mathcal{I}$ to $\mathcal{J}$, which are positive, to equal the total synaptic costs from $\mathcal{J}$ to $\mathcal{I}$, which are zero, and no solution exists to (S.8)—nor, by extension, to (21). As (21) is satisfied by all global minima of C on the task-preserving manifold, then no such minimum exists.

In the other direction, by Lemma 1, there exists some compact box $\mathcal{B} \in \mathbb{R}^N$ containing the sublevel set of C^0. Let $C^* = \inf\{C(\mathbf{h}) : \mathbf{h} \in \mathcal{B}\}$. As C is a continuous function on the compact set $\mathcal{B}$, there is some value $\mathbf{h}^* \in \mathcal{B}$ such that $C(\mathbf{h}^*) = C^*$. So $\mathbf{h}^*$ globally minimizes C. □

2.2 Global minima of the total cost are exponentially stable

We have shown conditions under which an optimal weight configuration $\mathcal{W}^*$ exists on the task-preserving manifold. We now argue, via an argument from strong convexity, that such a $\mathcal{W}^*$, when it exists, is uniquely determined and globally exponentially stable. Strong convexity is the property that the curvature of the objective function has a positive lower bound, and it implies that a minimum is unique and exponentially stable under gradient descent [3, Ch. 9]. Our approach is to re-parameterize the optimization problem and show that the cost function is strongly convex in the subspace of $\mathbb{R}^N$ that is relevant to synaptic balancing dynamics.

Proposition 2. *If a weight configuration $\mathcal{W}^*$ minimizes the total cost C on the task-preserving manifold, then (i) $\mathcal{W}^*$ is the unique weight configuration on the task-preserving manifold satisfying both* (21) *and* (S.5)*, and (ii) $\mathcal{W}^*$ is a globally exponentially stable equilibrium of synaptic balancing.*

Proof. Claims (i) and (ii) both follow by showing that for every initial weight configuration $\mathcal{W}^0$, the cost function C is strongly convex on a suitably defined re-parameterization of the task-preserving manifold, and that the dynamics of synaptic balancing is isomorphic to gradient descent dynamics in the strongly convex parameterization.

We begin with deriving a strongly convex formulation of the total cost. The $\{\mathbf{k}_i\}_{i=1}^K$ in (S.5) are mutually orthogonal, since they have non-overlapping nonzero

entries, so $\dim\{\mathbf{k}_i\}_{i=1}^K = \mathrm{K}$. Let $\mathbf{U}$ be any $(N-K)\times K$ matrix whose columns form an orthonormal basis for the orthogonal complement of $\mathrm{span}\{\mathbf{k}_i\}_{i=1}^K$. Then the trajectory of synaptic balancing is contained within the range of $\mathbf{U}$, i.e., $\mathbf{U}\mathbf{U}^T\mathbf{h}(t) = \mathbf{h}(t)$, and there exist reduced coordinates $\tilde{\mathbf{h}} \in \mathbb{R}^{N-K}$ satisfying

$$\tilde{\mathbf{h}}(t) = \mathbf{U}^T\mathbf{h}(t) \iff \mathbf{h}(t) = \mathbf{U}\tilde{\mathbf{h}}(t) \tag{S.9}$$

for all $t \geq 0$. We abuse notation slightly, writing $C(\mathbf{h})$ and $C(\tilde{\mathbf{h}})$ to refer to the total cost when considered as a function of, respectively, the original and reduced coordinates.

Suppose that a network has initial cost matrix $\mathbf{C}^0$. By assumption, a global minimum exists at $\mathcal{W}^*$, so by Prop. 1, the connected components of $\mathbf{C}^0$ are each strongly connected. Then by Lemma 1, some compact box $\mathcal{B} \subset \mathbb{R}^N$ exists such that the sublevel set $\{\mathbf{h} \in \mathbb{R}^N : C(\mathbf{h}) \leq C^0\}$ is contained in $\mathcal{B}$ for all $t \geq 0$. Then similarly $\{\tilde{\mathbf{h}} \in \mathbb{R}^{N-K} : C(\tilde{\mathbf{h}}) \leq C^0\} \subset \tilde{\mathcal{B}}$, where $\tilde{\mathcal{B}}$ is the projection of $\mathcal{B}$ onto the range of $\mathbf{U}$.

We will now show that C is strongly convex with respect to $\tilde{\mathbf{h}}$ on $\tilde{\mathcal{B}}$. This amounts to finding some $m > 0$ such that for every $\tilde{\mathbf{h}} \in \tilde{\mathcal{B}}$,

$$m \leq \min_{\tilde{\mathbf{u}} \in \mathbb{R}^{N-K}:\, \|\tilde{\mathbf{u}}\|=1} \tilde{\mathbf{u}}^T \frac{\partial^2 C}{\partial \tilde{\mathbf{h}}^2} \tilde{\mathbf{u}}. \tag{S.10}$$

The Hessian of C with respect to $\tilde{\mathbf{h}}$ is, via (S.9) and (34),

$$\begin{aligned}\frac{\partial^2 C}{\partial \tilde{\mathbf{h}}^2} &= \mathbf{U}^T \frac{\partial^2 C}{\partial \mathbf{h}^2}\mathbf{U} \\ &= p^2 \mathbf{U}^T\mathbf{L}\mathbf{U},\end{aligned}$$

where $\mathbf{L}$ is the Laplacian matrix associated with the graph with weights $\bar{\mathbf{C}} = \mathbf{C} + \mathbf{C}^T$.

A basic result on Laplacian matrices is that the null space of the Laplacian is the span of the indicator vectors of the connected components of the associated graph [4, Ch. 1]. In our case, this means that $\mathrm{span}\{\mathbf{k}_i\}_{i=1}^K$ is the (K-dimensional) null space of $\mathbf{L}(\tilde{\mathbf{h}})$ for all $\tilde{\mathbf{h}} \in \mathbb{R}^{N-K}$, since the task-preserving transformation does not alter network topology.

With these observations, the right hand side of (S.10) reduces, by the Courant-Fischer min-max theorem, to

$$\begin{aligned}&\min_{\tilde{\mathbf{u}} \in \mathbb{R}^{N-K}:\, \|\tilde{\mathbf{u}}\|=1} \tilde{\mathbf{u}}^T \frac{\partial^2 C}{\partial \tilde{\mathbf{h}}^2} \tilde{\mathbf{u}} \\ &\quad = p^2 \min_{\tilde{\mathbf{u}} \in \mathbb{R}^{N-K}:\, \|\tilde{\mathbf{u}}\|=1} \tilde{\mathbf{u}}^T\mathbf{U}^T\mathbf{L}\mathbf{U}\tilde{\mathbf{u}} \\ &\quad = p^2 \max_{U:\, \dim(U)=N-K} \; \min_{\mathbf{u} \in U:\, \|\mathbf{u}\|=1} \mathbf{u}^T\mathbf{L}\mathbf{u} \\ &\quad = p^2 \lambda_{K+1},\end{aligned} \tag{S.11}$$

and λ_n is the nth eigenvalue of $\mathbf{L}$, when ordered as $\lambda_1 \leq \lambda_2 \leq \ldots \leq \lambda_N$.

We have shown $\lambda_{K+1}(\mathbf{L}(\tilde{\mathbf{h}}))$ is positive for all values of $\tilde{\mathbf{h}}$. It remains to show that λ_{K+1} has a positive lower bound for all $\tilde{\mathbf{h}} \in \tilde{\mathcal{B}}$. This is immediate, however, from the fact that the eigenvalues $\{\lambda_i\}$, ordered by size as we have done here, are continuous functions of $\mathbf{L}$ [5, Ch. 1, §3], and $\tilde{\mathcal{B}}$ is compact: then λ_{K+1} must attain some minimum $\lambda^*_{K+1} > 0$ on $\tilde{\mathcal{B}}$. Plugging (S.11) into (S.10), we have

$$\begin{aligned}\min_{\tilde{\mathbf{u}} \in \mathbb{R}^{N-K}:\, \|\tilde{\mathbf{u}}\|=1} \tilde{\mathbf{u}}^T \frac{\partial^2 C}{\partial \tilde{\mathbf{h}}^2} \tilde{\mathbf{u}} &= p^2 \lambda_{K+1} \\ &\geq p^2 \lambda^*_{K+1}\end{aligned}$$

for all $\tilde{\mathbf{h}} \in \tilde{\mathcal{B}}$. So C is strongly convex with respect to the reduced coordinates $\tilde{\mathbf{h}}$ on the sublevel set of C^0.

Finally, we note that the linear relation (S.9) implies that the dynamics of gradient descent on C with respect to $\mathbf{h}$ is isomorphic to the dynamics gradient descent on C with respect to $\tilde{\mathbf{h}}$. Thus, although C is demonstrably *not* strongly convex with respect to the standard task-preserving manifold coordinates $\mathbf{h} \in \mathbb{R}^N$, synaptic balancing dynamics nonetheless inherits the desirable properties of gradient descent on a strongly convex function.

Because strongly convex functions possess a unique minimum which is exponentially stable under gradient descent, every global minimizer $\mathcal{W}^*$ of C on the task-preserving manifold is (i) the unique solution to (21) and (S.5) on the task-preserving manifold and (ii) globally exponentially stable under synaptic balancing dynamics. □

2.3 Normal matrices are balanced

As discussed in the main text, maximizing the robustness is equivalent to minimizing the total cost, which can be written as a matrix Frobenius norm:

$$C = \sum_{ij} \sigma_j^2 J_{ij}^2 = ||\mathbf{J\Sigma}||_F^2 \tag{S.12}$$

where we have introduced the diagonal matrix of moments $\Sigma = \mathbf{diag}\{\boldsymbol{\sigma}\}$. If $\mathbf{J\Sigma}$ is a normal matrix, then the cost (S.12) can be written in terms of its eigenvalues λ_j:

$$||\mathbf{J\Sigma}||_F^2 = \sum_j |\lambda_j|^2 \tag{S.13}$$

Now consider an arbitrary diagonal similarity transformation of the weight matrix $\mathbf{J}$, resulting in a potentially non-normal matrix $\widetilde{\mathbf{J\Sigma}} = \mathbf{Q}^{-1}\mathbf{JQ\Sigma}$. Since the diagonal matrices $\mathbf{Q}$ and $\mathbf{\Sigma}$ will commute, this transformation preserves the eigenvalues of $\mathbf{J\Sigma}$. If the matrix $\widetilde{\mathbf{J\Sigma}}$ has Schur decomposition with unitary matrix $\mathbf{U}$ and upper triangular matrix $\mathbf{\Lambda}$:

$$\widetilde{\mathbf{J\Sigma}} = \mathbf{U\Lambda U}^{-1}, \tag{S.14}$$

then we can see that the Frobenius norm and thus the cost C of this transformed matrix $\widetilde{\mathbf{J}\boldsymbol{\Sigma}}$ is equal to that of the matrix $\boldsymbol{\Lambda}$, which is always greater than or equal to the sum of the squared eigenvalues of the normal $\mathbf{J}\boldsymbol{\Sigma}$:

$$||\widetilde{\mathbf{J}\boldsymbol{\Sigma}}||_F^2 = ||\boldsymbol{\Lambda}||_F^2 \geq \sum_j |\lambda_j|^2 \tag{S.15}$$

since the eigenvalues of the matrix $\mathbf{J}\boldsymbol{\Sigma}$, $\widetilde{\mathbf{J}\boldsymbol{\Sigma}}$ and $\boldsymbol{\Lambda}$ are all shared. The inequality is saturated when the transformation $\mathbf{Q}$ is unitary. Therefore, we find that an arbitrary diagonal similarity transformation of the matrix $\mathbf{J}$ can not decrease the cost, in the case that $\mathbf{J}\boldsymbol{\Sigma}$ is normal.

Note that in the linear case, $\boldsymbol{\Sigma} = \mathbf{I}$ and this argument holds for arbitrary invertible matrices $\mathbf{Q}$ and normal weight matrices $\mathbf{J}$.

3 Bounds on minimum value

In general, we are not aware of an exact solution to the minimum value C^* of the total cost C of synaptic balancing. In this section, we calculate upper and lower bounds on C^* stated in Prop. 5. These bounds are computable based on the current state of the cost matrix, and so they help approximate the degree to which synaptic balancing will improve the robustness of a particular weight configuration.

3.1 Lower bound

We now state a basic bound on the synaptic costs that are attainable on the task-preserving manifold.

Lemma 2. *A synaptic cost matrix* $\mathbf{C}$ *evolving under synaptic balancing from an initial configuration* $\mathbf{C}^0$ *satisfies, for all pairs of neurons* i, j *and for all* $t \geq 0$,

$$\bar{c}_{ij}(t) \geq 2\hat{c}_{ij}, \tag{S.16}$$

where $\bar{c}_{ij}(t) = c_{ij}(t) + c_{ji}(t)$ (35) *and* $\hat{c}_{ij} = \sqrt{c^0_{ij}c^0_{ji}}$ (30).

Proof. For any two neurons i and j, the product of the reciprocal costs $c_{ij}c_{ji}$ is conserved by the task-preserving transformation. This product-conserving property follows directly from writing out the product of power-law costs (23) in terms of the task-preserving transformation (4):

$$\begin{aligned} c_{ij}c_{ji} &= c^0_{ij}c^0_{ji}e^{h_j-h_i}e^{h_i-h_j} \\ &= c^0_{ij}c^0_{ji}. \end{aligned} \tag{S.17}$$

To derive a general lower bound on $\bar{c}_{ij}$, let c^*_{ij} and c^*_{ji} be the optimizers of the two-variable minimization problem incorporating the product-conserving

constraint:

$$\begin{aligned} \text{minimize} \quad & c_{ij} + c_{ji} \\ \text{subject to} \quad & c_{ij}c_{ji} = c_{ij}^0 c_{ji}^0 \\ & c_{ij}, c_{ji} \geq 0. \end{aligned} \tag{S.18}$$

This, in turn, is equivalent to the single-variable problem

$$\begin{aligned} \text{minimize} \quad & c_{ij} + c_{ij}^0 c_{ji}^0 c_{ij}^{-1} \\ \text{subject to} \quad & c_{ij} \geq 0, \end{aligned} \tag{S.19}$$

which is convex. Analytically minimizing (S.19), we find that (S.18) has the unique solution

$$c_{ij}^* = c_{ji}^* = \sqrt{c_{ij}^0 c_{ji}^0} = \hat{c}_{ij}.$$

Thus, $\bar{c}_{ij} = c_{ij} + c_{ji} \geq c_{ij}^* + c_{ji}^* = 2\hat{c}_{ij}$ for all c_{ij}, c_{ji} accessible under the task-preserving transformation; in particular, the inequality holds along the trajectory of synaptic balancing. □

In short, the synaptic cost attains a minimum on the set of product-conserving matrices at the symmetric matrix whose elements are obtained by replacing each pair of initial reciprocal synaptic costs with its geometric mean.

As an additional consequence, we have that the geometric-mean matrix $\hat{\mathbf{C}}$ is in fact the equilibrium cost matrix of synaptic balancing whenever $\hat{\mathbf{C}}$ is accessible by the task-preserving transformation.

Lemma 3. *For all weight configurations on the task-preserving manifold, the total cost C satisfies the lower bound*

$$C \geq \sum_{ij} \hat{c}_{ij}. \tag{S.20}$$

If there is a weight configuration $\mathcal{W}^*$ on the task-preserving manifold whose cost matrix $\mathbf{C}^*$ is symmetric, then: $\mathbf{C}^* = \hat{\mathbf{C}}$; equality is attained in (S.20) *at $\mathbf{C}^*$; and $\mathcal{W}^*$ is a global minimizer of total cost on the task-preserving manifold.*

Proof. The total cost is $C = \sum_{ij} c_{ij} = \frac{1}{2}\sum_{ij} \bar{c}_{ij}$, and (S.20) is obtained by summing (S.16) over all synapses i, j. If there a a symmetric cost matrix $\mathbf{C}^*$ on the task-preserving manifold, then by the reciprocal-product-conserving constraint of the task-preserving transformation (S.17), it must be the geometric-mean matrix $\hat{\mathbf{C}}$. The remaining claims in the lemma follow immediately. □

Lemma 3 is incorporated in the main text as (42) in Prop. 5.

Next we derive an upper bound on the optimal value of the total cost, computable based on the current state of the cost matrix. Our approach is to bound the curvature of the sensitivity on the sublevel set of the initial total cost C^0 and then to minimize a quadratic upper envelope function using this bound.

Recall that $\frac{\partial^2 C}{\partial \mathbf{h}^2} = p^2 \mathbf{L}$ (34), i.e. the Hessian of the total cost takes the form of a Laplacian matrix whose elements are drawn from the conductance matrix $\bar{\mathbf{C}}$. (35). Let $\lambda_{\max}$ be the maximum eigenvalue of the Hessian. A simple upper bound is provided by Gershgorin's theorem:

$$\begin{aligned} \lambda_{\max} &\leq p^2 \max_i \sum_{j=1}^{N} |L_{ij}| \\ &= p^2 \max_i \left(|L_{ii}| + \sum_{j:j\neq i} |L_{ij}| \right) \\ &= p^2 \max_i \left(\sum_{j:j\neq i} \bar{c}_{ij} + \sum_{j:j\neq i} \bar{c}_{ij} \right) \\ &= 2p^2 \max_i \sum_{j:\, j\neq i} \bar{c}_{ij}. \end{aligned} \tag{S.21}$$

Since the maximum column sum of a matrix is upper bounded by the total sum of elements in the matrix, and in the case of $\bar{\mathbf{C}}$ that total sum decreases during gradient descent, we have:

$$\begin{aligned} \max_i \sum_{j:j\neq i} \bar{c}_{ij} &\leq \sum_{ij} \bar{c}_{ij} \\ &= 2C \\ &\leq 2C^0. \end{aligned} \tag{S.22}$$

This bound is too large by a factor of roughly N when synaptic costs are uniformly distributed throughout the network, but is (nearly) tight in the case of, for example, a hub-and-spoke network with a single central neuron projecting to all others, which each have a constant number of outgoing synapses.

Combining (S.21) and (S.22), we have in general that $\lambda_{\max} \leq M$ where

$$M = 4p^2 C^0. \tag{S.23}$$

We use the bound on the maximum eigenvalue to obtain an upper bound on C^*, the optimal value of the total cost. The fact that the curvature of C as a function of $\mathbf{h}$ is bounded implies that there exists a quadratic function $Q(\mathbf{h})$ tangent to S at $\mathbf{h} = 0$,

$$Q(\mathbf{h}) = C^0 + \mathbf{h}^T \left. \frac{\partial C}{\partial \mathbf{h}} \right|_{\mathbf{h}=0} + \frac{M}{2} \|\mathbf{h}\|_2^2 \tag{S.24}$$

such that $Q(\mathbf{h}) \geq C(\mathbf{h})$ for every $\mathbf{h}$ in the sublevel set of C^0 [3, §9.1.2, eq. 9.13]. This is known as a quadratic upper envelope function, and its minimum value Q^* is

$$Q^* = C^0 - \frac{1}{2M} \left\| \left. \frac{\partial C}{\partial \mathbf{h}} \right|_{\mathbf{h}=0} \right\|_2^2. \tag{S.25}$$

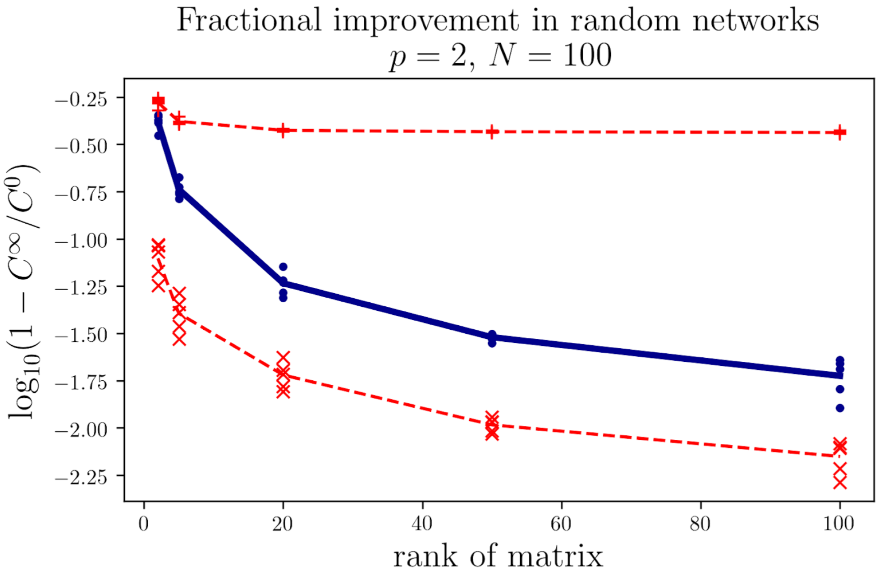

Fig. A: Fractional improvement in total cost for random networks as a function of connectivity matrix rank. Here we see that the improvement in cost $C^0 - C^\infty$ relative to the initial cost C^0, decreases as the rank of the matrix increases. Blue dots represent actual simulations; the red $+$ and $\times$ represent the upper and lower theoretical bounds presented in the preceding section.

Via the gradient descent rule (15), the initial gradient of the total cost is

$$\left.\frac{\partial C}{\partial h_k}\right|_{\mathbf{h}=0} = -p g_k^0. \tag{S.26}$$

By construction, the minimum of the upper envelope function upper bounds the minimum of C, i.e., $C^* \leq Q^*$. Plugging in (S.25), written in terms of (S.23) and (S.26), we obtain the bound

$$\begin{aligned} C^* &\leq Q^* \\ &= C^0 - \frac{1}{8C^0}\|\mathbf{g}^0\|_2^2, \end{aligned}$$

which is (41) in the main text.

The upper and lower bounds presented in this section are show in Fig. A as a function of the rank of the synaptic weight matrix. We can see that the relative improvement in total cost afforded by balancing decreases with increasing rank, suggesting that synaptic balancing is likely to be most effective for effectively low-dimensional networks.

4 Extension to positively-homogeneous nonlinearities

Here we detail an observation that the task-preserving transformation and our ability to minimize the sensitivity on the task-preserving manifold can be generalized to the case of the larger class of positively homogeneous nonlinearities.

A positively homogenous function ϕ of one variable x is one such that

$$\phi(sx) = s^k \phi(x) \tag{S.27}$$

for any $s > 0$ and $k \in \mathbb{R}$. The power of the scalar k is called the degree of homogeneity. This class of functions includes the monomials $\phi(x) = cx^k$ for some constant c. Note that the positively homogenous functions are a more general class of homogenous functions of degree k, which simply satisfy (S.27) with k an integer and $s \neq 0$.

Now we can revisit the task-preserving transformation, which we originally stated as $\mathbf{J} \mapsto e^{-\mathbf{H}}\mathbf{J}e^{\mathbf{H}}$. This transformation is a diagonal similarity transformation that preserves the RNN function exactly for homogenous nonlinearities of degree $k = 1$. Here we show that a similar transformation (but no longer a similarity transformation) extends this concept to more general positively homogenous nonlinearities.

Let us introduce a new transformation, $\mathbf{J} \mapsto e^{-\mathbf{H}}\mathbf{J}e^{k\mathbf{H}}$, with k a real scalar. The transformation of the input and output weights will be the same as before.

$$\pi_{\mathbf{h}}(\mathcal{W}) = (e^{-\mathbf{H}}\mathbf{W}^{\text{in}},\ e^{-\mathbf{H}}\mathbf{J}e^{k\mathbf{H}},\ \mathbf{W}^{\text{out}}e^{\mathbf{H}}). \tag{S.28}$$

The dynamics of the neural activity $\mathbf{x}$ in the network with transformed weights $\mathcal{W}$ are then

$$\mathbf{f}_{\mathcal{W}}(\mathbf{x},\mathbf{u}) = -\mathbf{x} + e^{-\mathbf{H}}\mathbf{J}^0 e^{k\mathbf{H}}\phi(\mathbf{x}) + e^{-\mathbf{H}}\mathbf{W}^{\text{in},0}\mathbf{u}. \tag{S.29}$$

Multiplying on the left by $e^{\mathbf{H}}$, and using that ϕ is positively homogeneous, we have

$$\begin{aligned} e^{\mathbf{H}}\mathbf{f}_{\mathcal{W}}(\mathbf{x},\mathbf{u}) &= -e^{\mathbf{H}}\mathbf{x} + \mathbf{J}^0\phi(e^{\mathbf{H}}\mathbf{x}) + \mathbf{W}^{\text{in},0}\mathbf{u} \\ &= \mathbf{f}_{\mathcal{W}^0}(\mathbf{z},\mathbf{u}), \end{aligned} \tag{S.30}$$

where $\mathbf{z} = e^{\mathbf{H}}\mathbf{x}$.

Now we can perform the same analysis as before to show that the time course of output neural activity is the same under $\mathcal{W}^0$ and $\mathcal{W}$. The time integral of the left hand side is the scaled neural activity of the network with weights $\mathcal{W}$:

$$\begin{aligned} \int_0^t e^{\mathbf{H}}\mathbf{f}_{\mathcal{W}}(\mathbf{x},\mathbf{u})\,\mathrm{d}t' &= e^{\mathbf{H}}\int_0^t \mathbf{f}_{\mathcal{W}}(\mathbf{x},\mathbf{u})\,\mathrm{d}t' \\ &= \tau e^{\mathbf{H}}\mathbf{x}(t), \end{aligned} \tag{S.31}$$

using an initial condition that $\mathbf{x}(0) = 0$.

The time integral of the right hand side is the neural activity of the network with weights $\mathcal{W}^0$:

$$\int_0^t \mathbf{f}_{\mathcal{W}^0}(\mathbf{z},\mathbf{u})\,\mathrm{d}t' = \tau\mathbf{x}^0(t). \tag{S.32}$$

Equating the left hand side and right hand side integrals, we find that

$$\mathbf{x}(t) = e^{-\mathbf{H}}\mathbf{x}^0(t), \tag{S.33}$$

The output neural activity is defined as

$$\mathbf{y} = \mathbf{W}^{\text{out}}\mathbf{x}., \tag{S.34}$$

which is (2) in the main text. As before, from (2) and (S.4), we have that the readout of the transformed network is

$$\begin{aligned} \mathbf{y}(t) &= \mathbf{W}^{\text{out}}\mathbf{x}(t) \\ &= (\mathbf{W}^{\text{out},0}e^{\mathbf{H}})(e^{-\mathbf{H}}\mathbf{x}^0(t)) \\ &= \mathbf{W}^{\text{out},0}\mathbf{x}^0(t) \\ &= \mathbf{y}^0(t). \end{aligned}$$

Therefore this modification to the TPT retains the task-preserving property of the original.

We must now check how the sensitivity transforms under this more general transformation. Eq. (9) in the main text tells us that the sensitivity can be written, with no assumptions on the nonlinearity $\phi(x)$, as

$$S = \sum_{ij} \sigma_j^2 J_{ij}^2 - 2 \sum_i \mu_i J_{ii} + N. \tag{S.35}$$

We need to check that the sensitivity is still a convex function on the task-preserving manifold for the more general case of $k \neq 1$. The elements of the connectivity matrix of the transformed network are

$$J_{ij} = J_{ij}^0 e^{kh_j - h_i}. \tag{S.36}$$

Above we saw that if $x_i^0(t)$ is the neural activity of hidden unit i for the original network under $\mathcal{W}^0$, then $x_i(t) = e^{-h_i} x_i^0(t)$ is the hidden unit neural activity under the transformed weights $\mathcal{W}$. In section the moments of the neural activity are constant with respect to the task-preserving transformation. However, we should no longer should this to be the case for more general positively-homogeneous nonlinearities. How then do μ_i and σ_i^2 transform with the new task-preserving transformation? We have

$$\begin{aligned} \mu_i =& \langle \phi'(x_i) \rangle = \langle \frac{d\phi(x_i)}{dx} \rangle \\ \sigma_i^2 =& \langle \phi'(x_i)^2 \rangle = \langle (\frac{d\phi(x_i)}{dx})^2 \rangle \end{aligned}$$

where

$$\begin{aligned} \frac{d\phi(x)}{dx} = \frac{\partial x_i^0}{\partial x_i} \frac{d\phi(x_i)}{dx_i^0} &= e^{hi} \frac{d\phi(e^{-h_i} x_i^0)}{dx_i^0} \\ &= e^{(1-k)h_i} \frac{d\phi(x_i^0)}{dx_i^0}. \end{aligned} \tag{S.37}$$

We can see that in homogenous case, corresponding to $k = 1$, we recover that $\frac{d\phi(x_i)}{dx_i} = \frac{d\phi(x_i^0)}{dx_i^0}$. Therefore we have

$$\mu_i = \langle \phi'(x_i) \rangle = e^{(1-k)h_i} \mu_i^0 \tag{S.38}$$

$$\sigma_i^2 = \langle \phi'(x_i)^2 \rangle = e^{2(1-k)h_i} (\sigma_i^0)^2 \tag{S.39}$$

indicating that these moments are no longer constant under the task-preserving transformation. Now we must check how the entire sensitivity (6) transforms.

Inserting (S.36), (S.38) and (S.39) into (6),

$$\begin{aligned} S =& \sum_{ij} e^{2(1-k)h_j} (\sigma_j^0)^2 (J_{ij}^0)^2 e^{2(kh_j - h_i)} \\ & - 2\sum_i e^{(1-k)h_i} \mu_i^0 J_{ii}^0 e^{h_i(k-1)} + N. \\ \implies S =& \sum_{ij} (\sigma_j^0)^2 (J_{ij}^0)^2 e^{2(h_j - h_i)} - 2\sum_i \mu_i^0 J_{ii}^0 + N. \end{aligned} \tag{S.40}$$

Happily, the terms depending on the degree of homogeneity k have cancelled, and we are left with the same sensitivity function as stated in the main text for the more restricted class of nonlinearities. If we define the synaptic costs in the same way as before, we have $c_{ij}^0 = (\sigma_j^0)^2 |J_{ij}^0|^2 \geq 0$, and a constant term $S_{\text{const}} = -2\sum_i \mu_i^0 J_{ii}^0 + N$. We then find as before,

$$S = \sum_{ij} c_{ij}^0 e^{2(h_j - h_i)} + S_{\text{const}} \tag{S.41}$$

for some constants $\{c_{ij}^0\}_{i,j=1}^N$, and S_{const}, where $c_{ij}^0 \geq 0$ for all i, j.

And again, as (S.41) is a positive linear combination, with constant coefficients, of exponentiated linear functions of $\mathbf{h}$ (which are convex), then it is in turn a convex function of $\mathbf{h}$.

The synaptic update rule for this generalization just requires a small modification in the updates to the recurrent weights:

$$\dot{J}_{ij} = J_{ij}(kg_j - g_i), \tag{S.42}$$

$$\dot{W}_{ik}^{\text{in}} = -W_{ik}^{\text{in}} g_i \tag{S.43}$$

$$\dot{W}_{kj}^{\text{out}} = W_{kj}^{\text{out}} g_j. \tag{S.44}$$

This treatment can be extended to apply to networks with positively homogeneous nonlinearities that vary from neuron to neuron by allowing the degree of the nonlinearity to depend on the neuron index, k_i.

References

[1] L. Hooi-Tong, "On a class of directed graphs?ith an application to traffic-flow problems," *Operations research*, vol. 18, no. 1, pp. 87–94, 1970.

[2] B. C. Eaves, A. J. Hoffman, U. G. Rothblum, and H. Schneider, "Line-sum-symmetric scalings of square nonnegative matrices," in *Mathematical programming essays in honor of George B. Dantzig Part II*, Springer, 1985, pp. 124–141.

[3] S. Boyd and L. Vandenberghe, *Convex optimization*. Cambridge university press, 2004.

[4] F. R. Chung and F. C. Graham, *Spectral graph theory*, 92. American Mathematical Soc., 1997.

[5] F. Rellich and J. Berkowitz, *Perturbation theory of eigenvalue problems*. CRC Press, 1969.
